# Microfluidic-based oral mucoadhesive nanozyme microspheres for immune modulation of xerostomia

**DOI:** 10.1016/j.mtbio.2026.102914

**Published:** 2026-02-10

**Authors:** Ye Fang, Xinyu Tao, Nengjie Yang, Jing Li, Jun Xiao, Liwei Qiu, Yujuan Zhu, Zhifeng Gu

**Affiliations:** Department of Rheumatology, Research Center of Clinical Medicine, Affiliated Hospital of Nantong University, Medical School of Nantong University, Nantong University, Nantong, 226001, China

**Keywords:** Xerostomia, Microspheres, Cerium oxide nanozyme, Mucoadhesive hydrogel, Saliva secretion

## Abstract

Xerostomia is defined as the clinical syndrome characterized by reduced salivary secretion and/or abnormal composition of saliva, with the essential pathological basis being salivary gland dysfunction. These symptoms could not be effectively and persistently alleviated using current therapies. This study presents a microfluidics-based oral adhesive nanozyme microsphere system for the treatment of xerostomia. The adhesive hydrogel coating on the outer layer of the microspheres can rapidly establish robust adhesion to the moist oral mucosa. Furthermore, it contains cerium oxide nanoparticles (CeNP) which would be slowly released in presence of saliva collagenase enzyme. Of particular interest are the remarkable anti-inflammatory and antioxidant properties of CeNP, which have demonstrated significant efficacy in neutralizing reactive oxygen species (ROS) produced by macrophages and markedly suppressing proinflammatory responses. Animal experiments have revealed this microsphere system can effectively alleviate the infiltration of inflammatory cells into the salivary glands and maintain the integrity of the salivary gland duct and acinus. Based on the transcriptomic analysis, adCe-MS demonstrates a robust therapeutic effect on mice with xerostomia by significantly downregulating key inflammatory pathways and chemokines while upregulating salivary secretion-related genes. These results suggest that this drug delivery system not only presents a novel strategy for mucosal nanomedicine administration but also offers an effective solution for the treatment of xerostomia.

## Highlights

• Integrated Functional Design: Combining microfluidic electrospray fabrication with nanoceria-alginate core and adhesive hydrogel coating to achieve simultaneous mucosal retention and controlled drug release.
• Pathology-Responsive Release: Enzymatically triggered CeNP delivery specifically under pathological collagenase-rich conditions.
• Dual Antioxidant-Anti-inflammatory Action: Cerium oxide nanoparticles provide simultaneous SOD/CAT-mimetic activities and anti-inflammatory effects.
• Mechanistic Validation: Transcriptomic profiling confirms dual modulation of inflammatory pathways and salivary secretion genes.

## Graphical abstract

**Figure:**
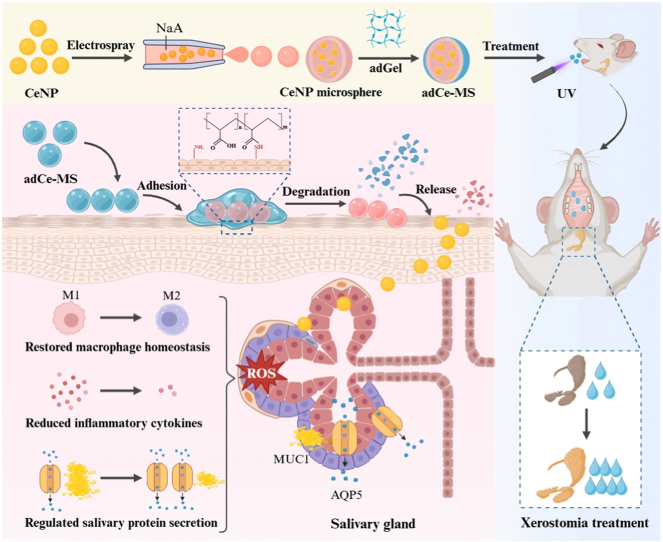

## 1 Introduction

Xerostomia is a prevalent clinical symptom resulting from salivary gland dysfunction, characterized by reduced or absent salivary secretion [[1], [2], [3], [4]]. The prevalence of xerostomia is approximately 20%-30% in the general population and can reach up to 40%-60% among the elderly and individuals with specific conditions (e.g., autoimmune diseases and head and neck cancer following radiotherapy) [[5], [6], [7]]. This condition not only induces dry oral mucosa, dysphagia, and dysgeusia but also leads to severe complications such as dental caries and oral infections, thereby significantly diminishing patients' quality of life and increasing societal medical burdens [8,9]. The etiology of xerostomia is multifactorial, primarily encompassing autoimmune factors (e.g., Sjögren's syndrome), drug-induced side effects (e.g., anticholinergic agents), and salivary gland damage caused by head and neck radiotherapy. Recent investigations have highlighted the important role of oxidative stress induced by reactive oxygen species (ROS) in salivary gland dysfunction, inducing glandular cell apoptosis and chronic inflammation, which further exacerbates disease progression [10]. Currently, clinical treatment modalities predominantly consist of artificial saliva substitution, cholinergic agonists (e.g., pilocarpine), and localized moisturizing therapies. Nevertheless, these approaches are constrained by limitations such as short duration of action, pronounced systemic adverse effects, or suboptimal patient adherence [[11], [12], [13]]. Despite the convenience of oral administration, the rapid clearance and insufficient targeting of drugs within the oral cavity severely impede their therapeutic efficacy [14]. It is therefore imperative to develop novel treatment modalities characterized by high efficiency, sustained efficacy, and precise regulatory capabilities. This concept has demonstrated potential in other areas of oral disease management. For instance, the topical application of biomaterials such as hyaluronic acid has proven effective in promoting oral mucosal healing by modulating the wound microenvironment [15].

**Fig. 1:**
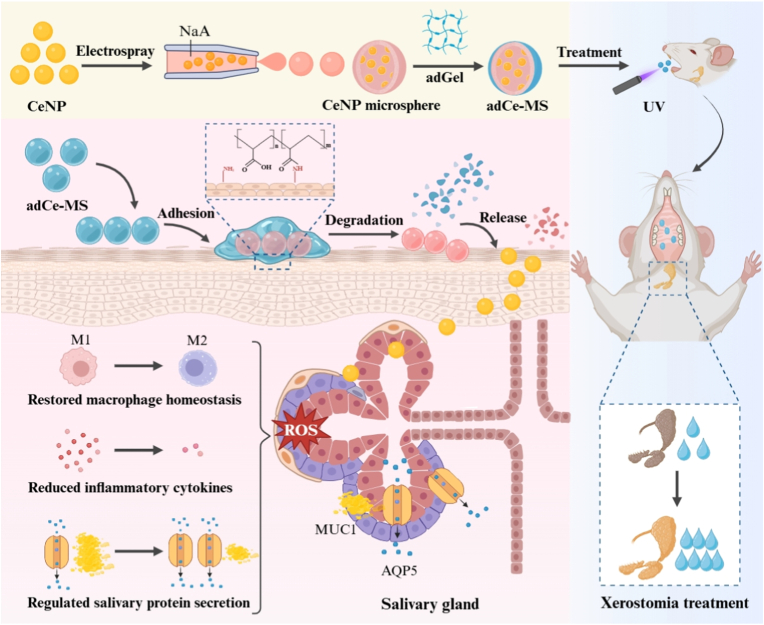
**The schematic illustration of the targeted micro-nano delivery system (adCe-MS) for treating xerostomia.** Cerium oxide nanoparticles (CeNP) were synthesized and then encapsulated into sodium alginate (NaA) microspheres using microfluidic electrospray technology, followed by oral adhesive hydrogel coating on the outer layer. In mouse models, this hierarchical microsphere system has demonstrated significant therapeutic efficacy, providing a novel strategy for the targeted treatment of xerostomia.

In this study, we developed oral mucoadhesive nanozyme microspheres as a novel therapeutic strategy for xerostomia (Fig. 1). As engineered nanomaterials, nanozymes possess the unique ability to mimic the catalytic properties of natural enzymes and have shown considerable translational potential in biomedicine [16]. Nanozymes can simulate the activities of natural enzymes, including peroxidase (POD) and superoxide dismutase (SOD) to precisely modulate redox homeostasis in vivo [17,18]. In contrast to conventional enzyme-based formulations, these engineered nanomaterials have improved structural stability, enhanced catalytic activity and can be delivered to specific sites by functionalization of their surfaces [19]. These advantages have made them versatile therapeutics with the potential to scavenge reactive oxygen species (ROS) in inflammatory diseases, generate toxic ROS against cancers, and demonstrate utility in antibacterial and neuroprotective applications [20,21]. Despite these promising properties, the effective delivery of nanomaterials, particularly across the oral mucosa, remains challenging. The main obstacles include the mucosal barrier, rapid clearance mechanisms, and enzymatic degradation, which collectively limit retention and bioavailability. Nanoparticle-based products that have high affinity and adhesion capacity toward the mucosa are currently under investigation as a potential strategy in order to address those challenges. Topical administration via the oral mucosa offers certain advantages for treating xerostomia, including targeted efficacy, minimized systemic exposure, and anatomical accessibility to salivary glands [22]. However, constant humidity, salivary flow, and the movement of tissues make it difficult to sustained mucosal drug delivery at the mucosal interface [23,24]. These constraints highlight the need for advanced mucoadhesive materials that maintain prolonged retention and controlled release under such dynamic conditions. Therefore, the rational design of mucoadhesive carriers that retain strong interfacial adhesion and preserve nanozyme catalytic activity in a wet and mobile oral environment is essential for achieving effective therapeutic outcomes [25].

Herein, we propose a microfluidics-derived adhesive micro-nano microsphere system integrated with nanozyme activity for immune modulation in xerostomia. This novel adhesive nanozyme-functionalized microsphere (adCe-MS), featuring a CeNP-loaded hydrogel core and a surface-adhesive coating, was successfully fabricated using microfluidic electrospray technology. adCe-MS possess excellent adhesion properties enabling its strong adhesion to different biological and synthetic surfaces. This durable mucosal adhesion enables sustained CeNP release in the oral microenvironment. In vitro experiments validated excellent biocompatibility, efficient ROS scavenging capacity, and significant anti-inflammatory activity that can effectively mitigate inflammatory responses induced by H_2_O_2_ in cellular models. In a murine model of Sjögren's syndrome, adCe-MS treatment markedly improved salivary flow rates, reduced inflammatory infiltration in major salivary glands, and ameliorated glandular structural damage, collectively alleviating xerostomia symptoms. Subsequent transcriptomic profiling of submandibular glands showed that adCe-MS not only suppressed critical inflammatory pathways (e.g., IL-17 and TNF signaling) and downregulated key pro-inflammatory genes, but also enhanced the expression of genes involved in salivary secretion and tissue regeneration. Collectively, this study establishes a promising locally-administrable immunomodulatory strategy with substantial translational potential for xerostomia treatment.

## 2 Results and discussion

**Fig. 2:**
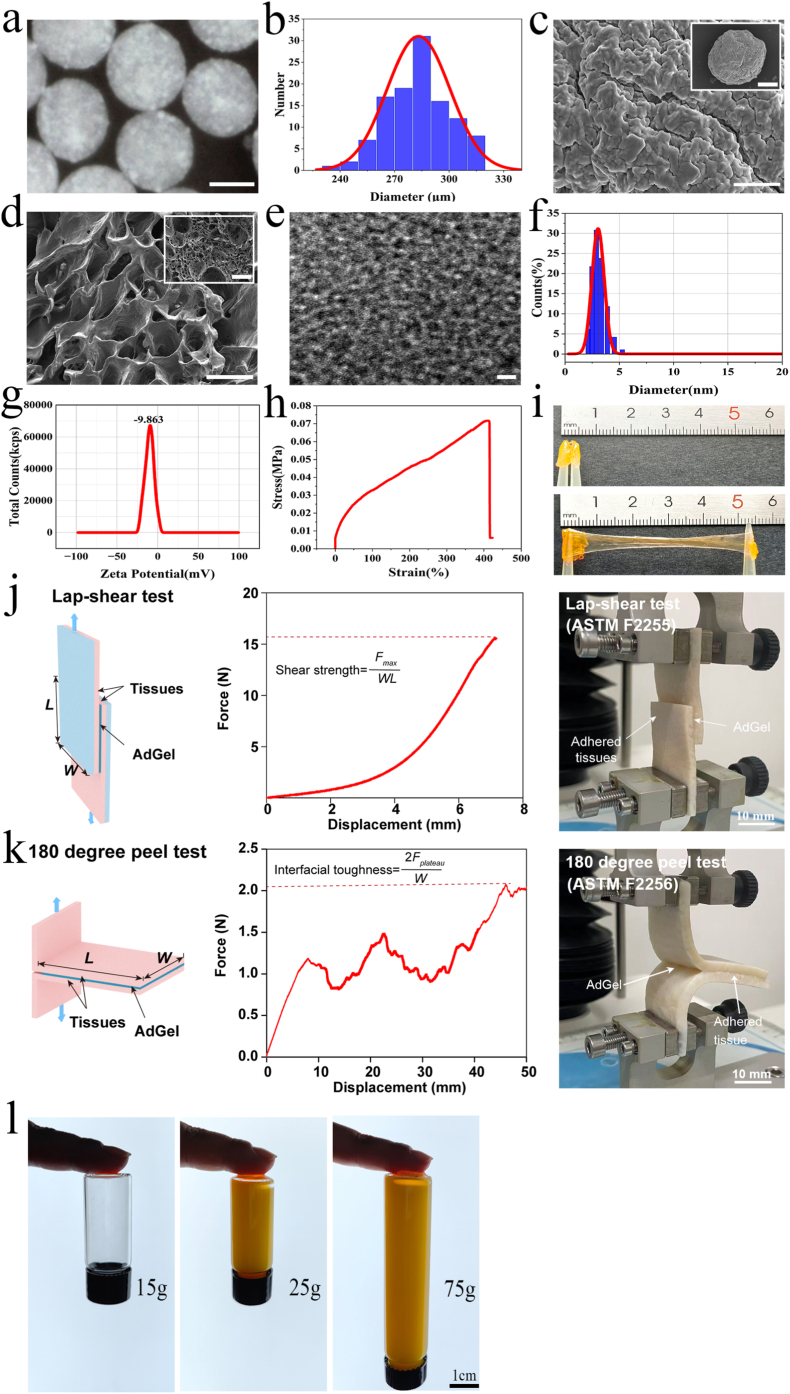
**Fabrication and characterization of mucoadhesive nanozyme-functionalized adCe-MS. a,** Bright-field image of adCe-MS. Scale bar, 150 μm** b,** Size distribution of the microspheres. **c,** Scanning electron microscopy (SEM) images of CeNP-NaA microspheres after freeze-drying. Scale bar, 50 μm (up), 5 μm (down). **d,** SEM images of adCe-MS. Scale bar, 250 μm (up), 50 μm (down). **e,** TEM image of CeNP. Scale bar, 10 nm. **f,** Hydrodynamic diameters distribution of CeNP in suspension. **g,** Zeta potential of CeNP. **h-i,** Adhesion properties of adGel. **j,** Shear strength measurement of adGel, following the standard lap-shear test (ASTM F2255). Scale bar, 10 mm. **k,** Interfacial toughness measurement of adGel**,** following the standard 180-degree peel test (ASTM F2256). Scale bar, 10 mm **l,** Tensile strength measurement of adGel. Scale bar, 1 cm. *F*, force; *F*_*max*_, maximum force (lap-shear test); *F*_*plateau*_, plateau force (peel test); *L*, length; *W*, width.

In a typical experiment, micro-nano hierarchical microspheres were fabricated based on microfluidic electrospray technology loaded with cerium oxide nanoparticles (CeNP) (Fig. S1) [26]. Specifically, these micro-nano hierarchical microspheres were simply prepared by external electrical stress, generating hundreds to thousands of uniform-sized and shaped microcapsules per minute, indicating that this system has great potential for the effective and high-throughput production of drug carriers for mucosal delivery. Oral mucosal delivery is a challenging but attractive therapeutic administration route that can circumvent both the harsh gastrointestinal environment and hepatic first-pass metabolism. To achieve immediate adhesion to wet tissue surfaces such as oral mucosa, a tissue-adhesive hydrogel structured as a dry double-sided tape (adGel) composed of gelatin and cross-linked polyacrylic acid grafted with *N*-hydroxysuccinimide ester was adopted [[27], [28], [29]]. To simulate the bio-inspired communication paradigm at the nanoscale, CeNP was loaded in the microspheres. The microspheres prepared by loading CeNP with sodium alginate (NaA) were uniform in size and shape, with a size of 200-350 μm (Fig. 2a–c). The encapsulation efficiency of CeNP in microspheres was 84.89% ± 2.93% (Fig. S2). Then the CeNP-NaA microspheres (CeNP-MS) were uniformly distributed in adGel by ultrasonic technology, effectively constructing nanozyme adhesive hydrogel microspheres (Fig. 2d). CeNP is a highly promising nanomaterial for therapeutic applications, with remarkable antioxidant, anti-inflammatory and immunomodulatory properties [30,31]. Transmission electron microscopy (TEM) showed that CeNP was dispersed and spherical in shape (Fig. 2e). And CeNP was characterized by uniform size, small particle size, negative Zeta potential (Fig. 2f–g), low cytotoxicity and easy endocytosis by cells. The adGel exhibits outstanding tissue adhesion and elastic deformation capabilities. The hydrogel displays remarkable elasticity and stretchability, with a maximum elongation at break exceeding 400% of its original length, indicating excellent mechanical resilience under tensile stress, which can simulate firm adhesion under the state of eating and chewing (Fig. 2h–i). In addition, direct adhesion tests showed that this hydrogel possesses a robust adhesion strength of at least 75 g (≈0.74 N), and can be stably fixed on various biological and synthetic surfaces (Fig. 2l). To further quantify its adhesive performance under simulated oral environmental conditions, lap-shear and 180-degree peel tests were performed on moist porcine skin. The adGel exhibited a high lap-shear strength of approximately 66.85 kPa and an interfacial toughness of about 173.32 J m^−2^, demonstrating strong and durable adhesion on wet tissues (Fig. 2j–k). Together, these results demonstrate that adGel is a highly adhesive hydrogel capable of maintaining stable adhesion under the dynamic and humid conditions characteristic of the oral cavity.

**Fig. 3:**
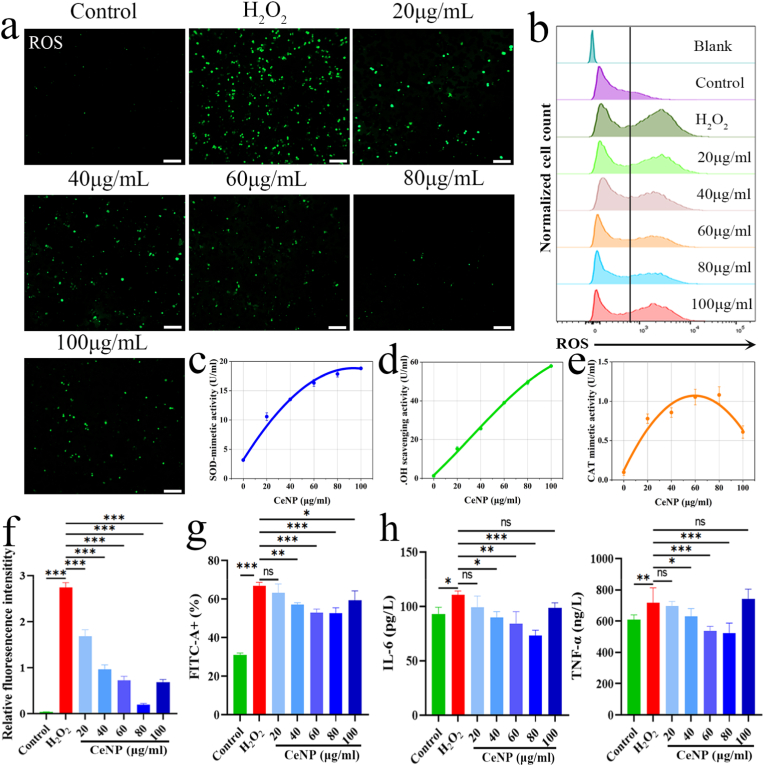
**Functional characterization of CeNP bioactivity. a,** Fluorescence images showing intracellular ROS signals by CeNP in H_2_O_2_-stimulated macrophages. Scale bar, 100 μm **b,** Flow cytometry measurement of intracellular ROS levels. **c,** SOD-mimetic activity of CeNP. **d,** Hydroxyl radical scavenging capacity of CeNP. **e,** Catalase-mimetic activity of CeNP. **f**, Quantitative analysis of ROS mean fluorescence intensity (MFI). **g**, Quantitative analysis of intracellular ROS levels measured by flow cytometry. **h,** Enzyme-linked immunosorbent assay (ELISA) measurements of cytokine (TNF-α and IL-6) levels in macrophage culture supernatants. All quantitative data are presented as mean ± SD (n = 3). *P < 0.05, **P < 0.01, ***P < 0.001.

CeNP encapsulated in the microsphere core is widely recognized for their multifunctional therapeutic potential, owing to their remarkable antioxidant, anti-inflammatory, and immunomodulatory properties [32,33]. To evaluate the biosafety of CeNP, we first performed in vitro viability assays using live/dead staining on macrophages exposed to CeNP. As shown in Fig. S3, CeNP exhibited excellent cytocompatibility in RAW264.7 macrophages, with cell proliferation rate exceeding 100% after 24-48 h of treatment at concentrations between 20 and 100 μg/mL, suggesting a favorable proliferative effect rather than merely an absence of toxicity. This favorable biosafety profile may be attributed to the small hydrodynamic size and moderate surface charge of CeNP, which mitigate nanoparticle-induced cytotoxicity. The antioxidant capacity of CeNP stems from their reversible Ce^3+^/Ce^4+^ redox cycling, which underlies their dual enzyme-mimetic activities [34,35]. CeNP functionally mimic both superoxide dismutase (SOD) and catalase (CAT), enabling efficient scavenging of superoxide anions and hydrogen peroxide, two major cellular ROS [36]. In vitro assays confirmed dose-dependent SOD-mimetic activity and hydroxyl radical scavenging capacity (Fig. 3c–d). CAT-like activity also increased concentration-dependently at lower concentrations (< 100 μg/mL) (Fig. 3e). The intracellular ROS-scavenging capacity of CeNP was further assessed in H_2_O_2_-stimulated macrophages. Upon H_2_O_2_ exposure, a marked increase in intracellular ROS levels was observed, confirming the successful induction of oxidative stress. Treatment with CeNP at concentrations below 60 μg/mL led to a dose-dependent reduction in ROS accumulation. In contrast, at higher concentrations (> 60 μg/mL), the ROS clearance efficiency of CeNP was notably attenuated (Fig. 3a–b, 3f–g). To evaluate the anti-inflammatory activity of CeNP, we analyzed the secretion of pro-inflammatory cytokines TNF-α and IL-6 in macrophages. H_2_O_2_ stimulation significantly upregulated the release of both cytokines, whereas CeNP treatment substantially suppressed their expression (Fig. 3h). These findings collectively indicate that CeNP mitigates oxidative stress and inflammatory activation through integrated antioxidant and anti-inflammatory mechanisms, highlighting their potential for modulating the tissue microenvironment in inflammatory settings.

**Fig. 4:**
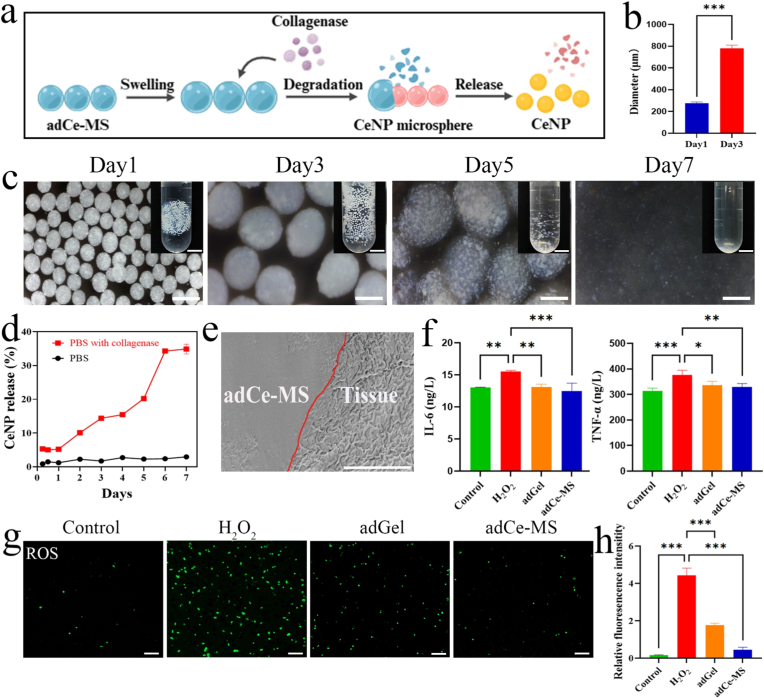
**In vitro functional evaluation and biocompatibility of adCe-MS. a**, Schematic illustration of the enzymatic degradation process of adCe-MS. **b,** Size variation of microspheres during degradation. **c,** Morphological changes of adCe-MS upon sequential incubation in collagenase-containing PBS. Scale bar, 5 mm (right), 500 μm (left). **d,** Cumulative release of CeNP from adCe-MS in PBS with or without collagenase. **e,** SEM image demonstrating the adhesive interface between adCe-MS and murine tongue tissue (red line indicates integration boundary). Scale bar, 10 μm. **f,** ELISA quantification of the amount of TNF-α and IL-6 released into cell culture supernatant. **g,** ROS scavenging activity of adGel and adCe-MS. Scale bar, 100 μm **h,** Quantitative analysis of ROS MFI. All quantitative data are presented as the means ± SD (n = 3). *P < 0.05, **P < 0.01, ***P < 0.001.

Next, the biocompatibility of adGel and adCe-MS was evaluated using live/dead staining in macrophages. No significant reduction in cell viability was observed following treatment with varying concentrations of adGel and adCe-MS, indicating that their favorable biosafety profile (Fig. S4a–b). Given that salivary collagenase levels, particularly matrix metalloproteinase MMP-9, are elevated in xerostomia conditions such as Sjögren's syndrome, we investigated the CeNP release profile under physiologically relevant environments [37]. The cumulative release of CeNP was monitored in both collagenase-containing buffer (mimicking the pathological oral milieu) and enzyme-free buffer (simulating normal conditions). The results demonstrated that adCe-MS degraded completely within approximately 7 days (Fig. 4a–d), enabling sustained CeNP release. The controlled-release profile of this system may reduce the frequency of administrations and improve clinical adherence. We further evaluated the in vitro bioactivity of the adCe-MS system. Exposure to adCe-MS-conditioned media significantly attenuated intracellular ROS generation in macrophages (Fig. 4g–h) and suppressed the secretion of the pro-inflammatory cytokines TNF-α and IL-6 (Fig. 4f). Interestingly, it was found that the hydrogel matrix itself (adGel) exhibited comparable anti-inflammatory and antioxidant activity in these short-term experiments. This effect was likely attributable to the release of bioactive gelatin-derived peptides during hydrogel hydration, particularly those containing RGD motifs [38]. As intrinsic components of the gelatin and gelatin methacrylate (GelMA) used in the formulation, these peptides have been well documented to modulate macrophage inflammatory responses [39,40]. The scanning electron microscopy (SEM) image also demonstrated that adCe-MS could adhere tightly on murine tongue tissues (Fig. 4e), suggesting the effective retention in the oral cavity. In conclusion, the adCe-MS integrates strong mucoadhesion, excellent biocompatibility, sustained drug release, and potent anti-inflammatory and antioxidant activity, highlighting its promise as a localized therapeutic strategy for xerostomia.

Before assessing the in vivo distribution and therapeutic efficacy of adCe-MS, we first evaluated the biocompatibility of its application process, which entails brief in-situ UV curing. Human epidermal cells (HaCaT) and murine oral mucosa were irradiated by UV light (284 nm, 10 W) for normal duration of applications (5-10 s). The live/dead cell staining and histological analysis demonstrated that this exposure did not induce detectable cytotoxicity or tissue damage (Fig. S5). Then we compared the pharmacokinetics of our localized mucosal delivery approach against systemic gavage. Ex vivo fluorescence imaging at 6, 12, and 24 h after the application of Cy5-labeled formulations revealed that topical application of Cy5-adCe-MS resulted in significantly stronger and more sustained fluorescence signals specifically in the salivary glands compared to gavage delivery (Fig. S6). The fluorescence signal of the topical adCe-MS group was still intense at 24 h, but the fluorescent intensities declined rapidly after 6 h and could hardly be detected at 24 h in the gavage groups. To evaluate long-term biosafety, H&E staining has been performed by major organs (heart, liver, spleen, lungs, and kidneys) from adCe-MS-treated mice, which showed no significant morphological abnormalities compared to PBS controls (Fig. S7a). Moreover, serum levels of hepatic enzymes including alanine aminotransferase (ALT), aspartate aminotransferase (AST), the ALT/AST ratio, and the renal function marker blood urea nitrogen (BUN) revealed no statistically significant differences relative to the PBS-treated group (Fig. S7b). Overall, these results demonstrated that the adCe-MS system can be safely administrated and achieve superior localized enrichment in the target tissue, which proved its potential as an effective local strategy for managing xerostomia.

**Fig. 5:**
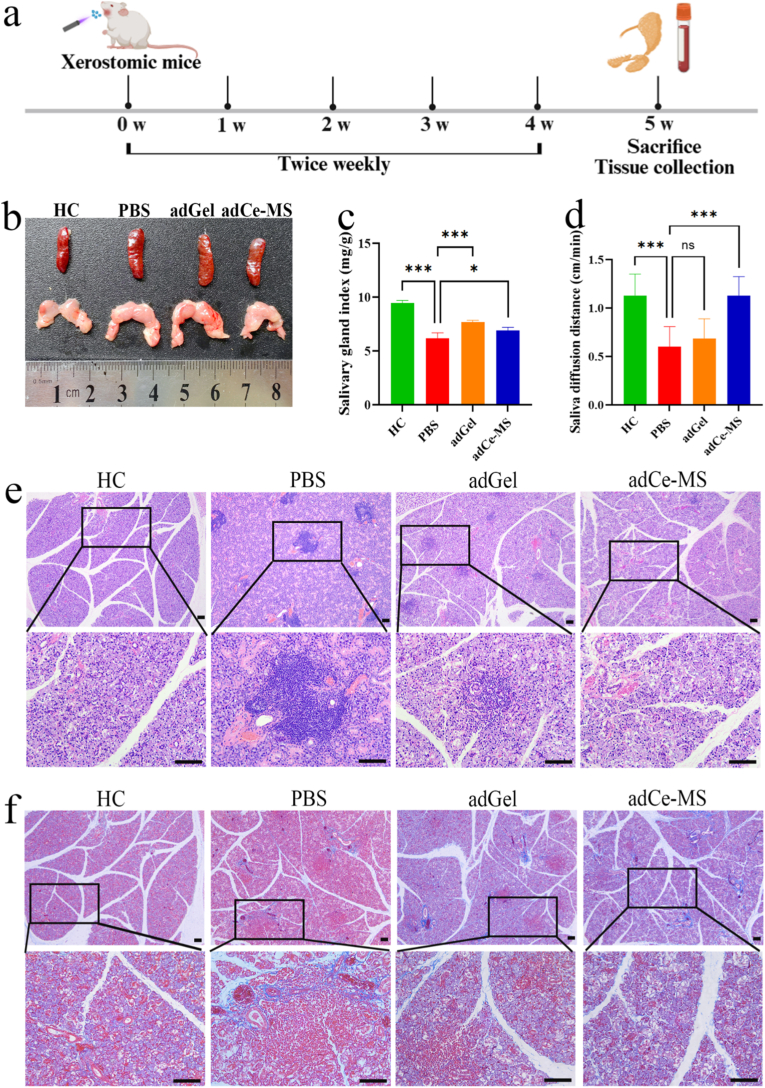
**In vivo therapeutic efficacy of adCe-MS in a murine model of xerostomia. a,** Schematic timeline of the in vivo experimental design. **b,** Representative gross images of the spleen and salivary glands from the different treatment groups. **c,** Salivary gland index across experimental groups (n = 4). **d,** Measurement of stimulated saliva secretion across treatment groups (n = 6). **e,** Representative H&E-stained sections of submandibular glands from different groups. Scale bar, 100 μm. **f,** Representative Masson-stained sections of submandibular glands from different groups. Scale bar, 100 μm. All quantitative data are presented as the means ± SD (n = 3). *P < 0.05, **P < 0.01, ***P < 0.001.

Having established the biofunctional performance of adCe-MS in vitro, we next evaluated its therapeutic efficacy in a xerostomia mouse model (NOD/LtJ strain) using the experimental timeline outlined in Fig. 5a. Body weight monitoring revealed no significant differences between PBS-treated and adCe-MS-treated xerostomic mice throughout the study period (Fig. S8a), confirming that the mucoadhesive microspheres did not interfere with normal feeding behavior or cause systemic toxicity. It should be noted that the healthy control C57BL/6 mice exhibited lower baseline body weights compared to NOD/LtJ mice, consistent with established strain characteristics and unrelated to experimental interventions. Notably, adCe-MS treatment induced significant modulations in organ indices. We observed a marked reduction in spleen index coupled with an increased salivary gland index in the adCe-MS group compared to PBS controls (Fig. 5b–c, S8b), suggesting potential attenuation of systemic immune activation and concurrent protection against salivary gland dysfunction. To better verify the therapeutic effect, we collected saliva samples using absorbent paper and evaluated them from various groups. The results revealed a marked increase in saliva secretion in the adCe-MS group relative to the PBS controls (Fig. 5d). Accordingly, we focused our subsequent detailed histological analyses on the submandibular glands, as they are the major saliva-producing organs in mice [41]. Then, these glands of each group were stained with HE and Masson for histological identification (Fig. 5e–f). In the PBS-treated group, the submandibular glands showed disordered glandular structure, replaced by fat vacuoles and pink fibrous tissue, with multifocal lymphocytic infiltration in the interstitium, atrophy of acinar cells, and reduced ducts. Notably, the adCe-MS group exhibited a significant resolution of damage, with salivary gland morphology closely resembling that of healthy tissue.

**Fig. 6:**
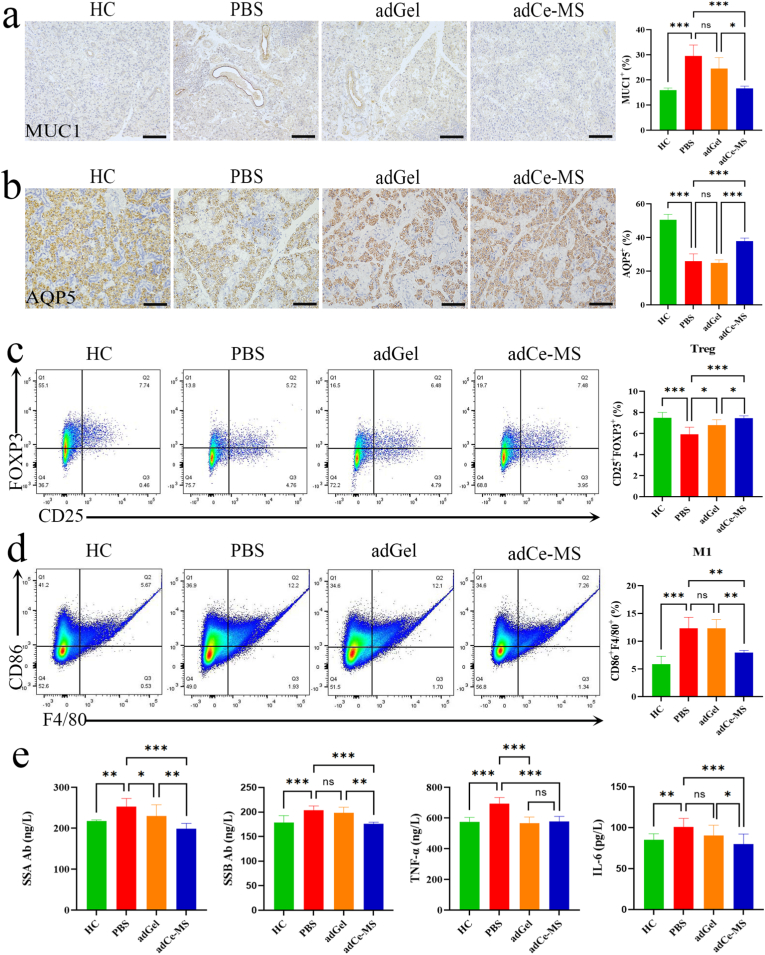
**Systemic immunomodulatory effects and functional restoration of submandibular glands following adCe-MS treatment in xerostomic mice. a-b,** Immunohistochemical analysis of MUC1 (a) and AQP5 (b) expression in submandibular gland tissues (n = 4). Scale bar, 100 μm. **c,** The proportion of Treg cells (CD25^+^ FOXP3+) in spleen by flow cytometry (n = 8). **d,** Proportions of M1-polarized macrophages (F4/80^+^CD86^+^) in the spleen, as determined by flow cytometric analysis. (n = 8). **e,** Plasma levels of autoantibodies (SSA-Ab, SSB-Ab) and pro-inflammatory cytokines (TNF-α, IL-6) as measured by ELISA (n = 8). Data are represented as means ± SD. *P < 0.05, **P < 0.01, ***P < 0.001.

In the NOD/LtJ mouse model of Sjögren's syndrome, aquaporin 5 (AQP5) and mucin 1 (MUC1) are key molecules for maintaining salivary gland function and pathological damage, and their expression changes directly reflect glandular secretion dysfunction and immune damage [42,43]. Tissue staining of submandibular glands with 3,3′-Diaminobenzidine (DAB) and hematoxylin further confirmed that the abnormal expression of MUC1 was reduced and mucus secretion was restored under adCe-MS intervention; the expression of AQP5 increased, enhancing the secretion of the aqueous phase in saliva (Fig. 6a–b), reversing mucus dysfunction. Overall, adCe-MS achieved significant therapeutic effects. Next, based on the above observations, we conducted more detailed studies. Regulatory T (Treg) cells are critically involved in the pathogenesis of Sjögren's syndrome. A reduction in their number or functional defect can lead to the breakdown of immune tolerance, promoting glandular damage and systemic autoimmune responses. Flow cytometry indicated that the proportion of Treg cells (CD25^+^ FOXP3^+^) in the spleen of mice treated with adCe-MS was significantly increased (Fig. 6c). Meanwhile, macrophages, particularly the M1 phenotype, are essential for regulating inflammatory responses and are closely associated with the autoimmune pathogenesis of Sjögren's syndrome. To examine the effect of adCe-MS on macrophage activation, we used flow cytometry and found that the proportion of M1 macrophages (F4/80^+^CD86^+^) in the spleen was significantly reduced after adCe-MS treatment compared to the PBS group and adGel group (Fig. 6d). Consistent with the above results, ELISA analysis demonstrated that plasma concentrations of autoantibodies (anti-SSA/Ro and anti-SSB/La) and pro-inflammatory cytokines (IL-6 and TNF-α) were significantly lower in the adCe-MS group compared to the PBS group. Meanwhile, this reduction remained statistically significant compared to the adGel group for anti-SSA/Ro, anti-SSB/La and IL-6 (Fig. 6e). All data indicate that adCe-MS have a significant therapeutic effect on xerostomia by regulating tissue remodeling and inflammatory cell infiltration.

To further elucidate transcriptomic alterations in the salivary glands of xerostomic mice, RNA sequencing (RNA-Seq) was performed on submandibular glands from PBS-treated mice and healthy controls. Principal coordinate analysis (PCoA) revealed clear separation between the groups, reflecting substantial differences in global gene expression profiles (Fig. S9a). Differential expression analysis identified a total of 7244 differentially expressed genes (DEGs), among which 7164 were upregulated and 80 were downregulated in the PBS-treated group compared with the healthy controls (Fig. S9b). Gene ontology (GO) enrichment analysis indicated that these DEGs were predominantly related to biological processes such as immune response, response to stimuli, and metabolic activities (Fig. S9c). Moreover, heatmap visualization of inflammation-related genes demonstrated significant upregulation of Chemokines (*CCL12, CCL20, CXCL1,* and *CXCL10*), members of the tumor necrosis factor receptor superfamily (*TNFRSF14* and *TNFRSF17*) and so on in the PBS-treated group. These results demonstrate that there is an intense inflammatory environment in the submandibular glands in murine models of xerostomia (Fig. S9d). Collectively, these results provide a molecular rationale for glandular dysfunction observed in this disease model and highlight key signaling pathways that may serve as potential targets for therapeutic intervention.

**Fig. 7:**
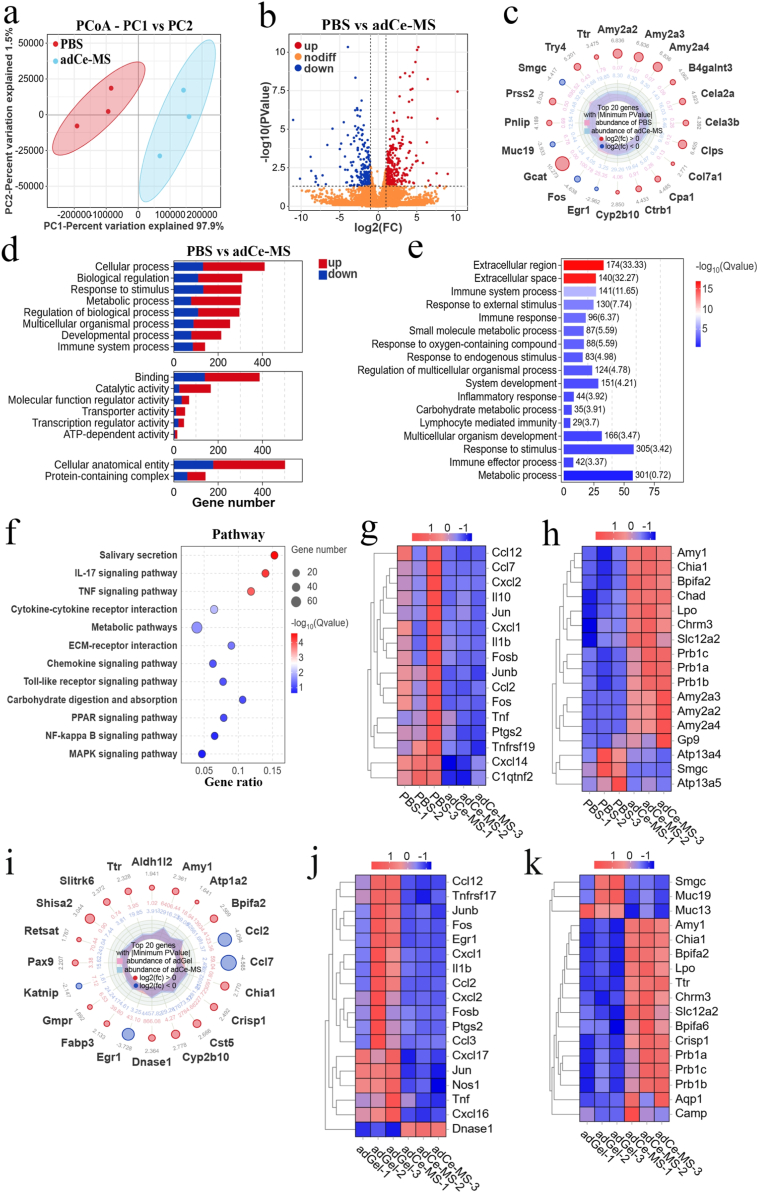
**Transcriptomic profiling reveals molecular mechanisms underlying adCe-MS-mediated recovery in xerostomic mice. a,** Principal coordinate analysis (PCoA) of submandibular gland transcriptomes from adCe-MS- and PBS-treated groups. **b,** Volcano plots of DEGs between PBS- and adCe-MS groups. **c,** Radar map showing the top 20 most significantly regulated DEGs between PBS and adCe-MS groups. **d,** Gene Ontology (GO) functional classification of DEGs **e,** GO enrichment analyses of DEGs. **f,** KEGG pathway enrichment analysis of DEGs. **g,** Heatmap visualization of inflammation-related gene expression in PBS vs adCe-MS groups. **h,** Heatmap of salivary secretion-related gene expression in PBS vs adCe-MS groups. **i,** Radar chart of the top 20 most significant DEGs between adGel and adCe-MS groups. **j,** Heatmap of inflammation-related gene expression in adGel vs adCe-MS groups. **k,** Heatmap of salivary secretion-related gene expression in adGel vs adCe-MS groups.

We next investigated whether the adCe-MS treatment could reverse these changes in gene expression. PCoA showed distinct clustering of the adCe-MS group compared to PBS controls (Fig. 7a), indicating global transcriptomic remodeling. Comparative analysis identified 554 DEGs (342 up-regulated, 212 down-regulated) in adCe-MS-treated glands (Fig. 7b). A radar plot of the top 20 most significant DEGs (adjusted *P* < 0.05 and |log2FC| ≥ 2.0) revealed changes in salivary secretion-related genes (*AMY*, *TRY*, *CLP*, *MUC*, and *SMGC*), and oxidative stress/inflammation-associated genes (*FOS*, *EGR*) (Fig. 7c). GO enrichment analysis showed predominant involvement in response to stimulus, metabolic processes, and immune system processes (Fig. 7d–e), suggesting adCe-MS modulates immune-inflammatory networks. Kyoto Encyclopedia of Genes and Genomes (KEGG) pathway analysis further indicated enrichment in salivary secretion pathways, IL-17 signaling, and TNF signaling (Fig. 7f), pathways critically associated with glandular function and inflammation. To further investigate the impact of adCe-MS on inflammation, alterations in inflammation-related genes were visualized using a heatmap. As shown in Fig. 7g, adCe-MS significantly reduced the expression levels of chemotactic factors such as *CCL2*, *CCL7*, *CCL12*, *CXCL1*, *CXCL2*, and *CXCL14*, as well as key inflammatory mediators including *TNF*, *TNFRSF19*, *IL-1β*, *FOS*, *FOSB*, *JUN*, *JUNB*, and *PTGS2*. These results indicated that adCe-MS effectively attenuated oxidative stress and inflammatory processes in the submandibular glands of xerostomic mice. In parallel, heatmap analysis of genes associated with saliva secretion revealed that adCe-MS treatment upregulated amylase family (*AMY2A2*, *AMY2A3*, *AMY2A4*), proline-rich protein gene family (*PRB1A*, *PRB1B*, *PRB1C*) which are known to contribute to oral protective mechanisms, and LPO. This suggested a potential role of adCe-MS in promoting salivary secretion and protecting salivary gland tissue from damage (Fig. 7h). Collectively, transcriptomic analysis revealed that adCe-MS mitigated submandibular gland injury, enhanced salivary production, and restored oral lubrication through the inhibition of oxidative stress and inflammatory responses.

To precisely delineate the specific therapeutic contribution of the CeNP nanozyme independent of the mucoadhesive hydrogel carrier, a comparative transcriptomic analysis was performed between the adGel-treated and adCe-MS-treated groups. PCoA and volcano plots revealed a distinct separation in global gene expression profiles between the two groups, indicating that the incorporation of CeNP induces significant transcriptomic alterations beyond those attributable to the adhesive hydrogel matrix alone (Fig. S10a and c). GO enrichment analysis of DEGs demonstrated their predominant association with biological processes such as response to stimuli, metabolic processes, and immune system regulation (Fig. S10b and d). Further examination of the most significantly altered genes highlighted a unique gene expression signature associated with nanozyme activity (Fig. 7i). Notably, heatmap analysis showed that adCe-MS treatment led to a more pronounced downregulation of critical pro-inflammatory mediators, including *TNF*, *IL-1β*, *FOS*, *JUN*, and multiple chemokines such as *CCL2* and *CXCL1*, compared to the adGel group (Fig. 7j). This indicates that the integration of CeNP into the adhesive system confers superior anti-inflammatory activity. Furthermore, genes associated with salivary secretion—such as amylase family members (*AMY1*) and proline-rich protein gene family (*PRB1A*, *PRB1B*, *PRB1C*)—were significantly upregulated in the adCe-MS group compared with the adGel group. Meanwhile, the expression of Mucins (*MUC13* and *MUC19*) was obviously lower than that of adGel group (Fig. 7k). Collectively, these results demonstrate that the enhanced therapeutic efficacy of adCe-MS is specifically mediated by the CeNP, which acts synergistically with the hydrogel delivery system to robustly suppress inflammatory pathways and actively promote transcriptional programs essential for salivary gland functional recovery.

## 3 Conclusion

In this study, we have developed a microfluidic-based oral mucoadhesive nanozyme microsphere (adCe-MS) that represents a paradigm shift in the management of Sjögren's disease-related xerostomia. By integrating a tissue-adhesive hydrogel with CeNP, this system enables sustained, localized therapy directly within the oral cavity. While the adhesive hydrogel carrier ensures localized delivery and may provide inherent biocompatibility, the embedded CeNP nanozymes are primarily responsible for the therapeutic efficacy. Our findings demonstrate that adCe-MS effectively mitigates the core pathological drivers of xerostomia associated with Sjögren's syndrome: it scavenges reactive oxygen species to alleviate oxidative stress and robustly suppresses pro-inflammatory responses, leading to reduced immune cell infiltration and the preservation of salivary gland architecture. Crucially, transcriptomic analysis confirmed that this therapeutic efficacy stems from a dual mechanism, simultaneously inhibiting key inflammatory pathways and upregulating genes critical for salivary secretion. This coordinated action resulted in significantly increased saliva flow and restored oral lubrication in a xerostomic mouse model. Therefore, this work moves beyond palliative care and establishes a targeted, mechanism-based strategy that directly interrupts the immune-oxidative cascade responsible for glandular damage.

adCe-MS also has potential for modulating the pathological oral microenvironment in xerostomia where decreased salivary flow results in a pro-inflammatory and oxidative microenvironment that causes oral microbial dysbiosis and aggravates tissue damage [44]. Within this system, CeNP might be able to reverse these processes in two ways. It can directly scavenge ROS and suppress inflammation to ameliorate the underlying microenvironment, while its intrinsic antibacterial and anti-biofilm activities are able to suppress the overgrowth of pathogenic microorganisms [[45], [46], [47]]. Compared to other nanozymes investigated for anti-inflammatory therapy, such as platinum-based nanozymes with high production costs or manganese oxide nanozymes that would require further toxicological studies before clinical application, CeNP offers an overall and integrative advantage [48,49]. It is characterized by adaptive multi-enzyme-mimetic activity, inherent antimicrobial properties, and well-established biocompatibility [50,51]. In conclusion, the unique properties of CeNP allow this nanomaterial to be used as a highly effective and specific system to manage the oral inflammation through a local and prolonged delivery of therapeutics.

The design of adCe-MS presents several distinct advantages over other advanced microsphere systems. In contrast to systems that require injectable delivery, such as intra-articular injection of MXene-containing hydrogel microspheres in the treatment of osteoarthritis [52], adCe-MS employs a non-invasive, mucoadhesive strategy for localized delivery. This approach can both enhance the residence time in the targeted area and circumvent issues related to injection-associated discomfort, potential infection, and patient compliance [53]. Moreover, the release profile of adCe-MS is specially designed to respond to locally increased enzymatic activity within the pathological microenvironment, which would be an important advance compared with systems reliant on externally applied physical stimuli. Beyond serving as a passive vehicle for therapeutic agents, such as anti-inflammatory drugs or growth factors, adCe-MS acts as an active therapeutic platform [54]. Its nanozyme core actively modulates redox homeostasis through catalytic activity, thereby targeting underlying disease mechanisms rather than merely delivering pharmacological payloads. Altogether, the adCe-MS platform represents a promising and translatable strategy for the local management of xerostomia and potentially other oral inflammatory diseases. Future work will focus on long-term biosafety evaluation, scaling-up production, and exploring its applicability in clinical settings.

## 4 Methods

### 4.1 Materials

Acrylic acid, gelatin methacrylate (GelMA; type A bloom 90-100 from porcine skin with 60% substitution), acrylic acid *N*-hydroxysuccinimide ester (AAc-NHS ester), *α*-ketoglutaric acid, gelatin (type A bloom 300 from porcine skin), sodium alginate (NaA), cerium (III) nitrate hexahydrate (99% trace metals basis) and disodium citrate (BioXtra, anhydrous, ≥99.5%) were obtained from Sigma-Aldrich (USA). Ammonia solution and anhydrous calcium chloride (AR ≥96%) were obtained from Aladdin (China).

### 4.2 Preparation of adGel

The adGel formulation was prepared by dissolving the following components in deionized water: acrylic acid (30% w/w), gelatin (10% w/w), AAc-NHS ester (1% w/w), GelMA (0.1% w/w), and α-ketoglutaric acid (0.2% w/w). After stirring for 12 h, the solution was passed through a 0.45 μm sterile syringe filter to obtain this viscous hydrogel. To facilitate the visualization of adGel, we added red ink (for photos) to it before curing.

### 4.3 Mechanical tests

For direct adhesion tests, 50 μL of UV-cured adGel was applied to one fingertip and sequentially loaded into glass vials of different weights (15-75 g) to assess the hydrogel adherence over 2 min; A volume of 50 μL of adGel was cured and attached to 200 μL of the gun tip, and its maximum elongation was measured through uniform stretching; For tensile tests, the adGel was further cured and cut to produce standard specimens of 20 × 5 mm^2^ (n = 5), and their mechanical properties were evaluated by generating stress-strain curves using an electronic universal material testing machine (Instron 5944, USA).

Lap-shear and 180-degree peel tests were conducted on moist porcine skin strips (1-2 mm thick) using a universal testing machine (TA-XTplus, Xiamen Chaoji Instrument Equipment Co., Ltd., China). For lap-shear tests, 200 μL adGel was applied on a 10 mm × 25 mm overlap region, UV-cured, and bonded to a second strip. The assembly was stretched at 1 mm/min until failure. For 180-degree peel tests, 1000 μL adGel was applied over 50 mm × 25 mm, cured, and peeled at 180-degree under the same speed. Lap-shear strength (kPa) and interfacial toughness (J·m^−2^) were derived from force-displacement curves.

### 4.4 Preparation of CeNP and the CeNP-NaA microspheres (CeNP-MS)

CeNP was prepared via a two-step aqueous precipitation route. 108.5 mg cerium nitrate hexahydrate (Solution A) and 100 mg disodium citrate (Solution B) were dissolved in 2 mL and 1 mL deionized water, respectively. After mixing, 50 mL 0.4 M ammonia solution was added with 24 h stirring. The mixture was centrifuged (2600×*g*, 30 min), dialyzed (3000 Da, 24 h), re-centrifuged (15000×*g*, 10 min), filtered (0.22 μm), and freeze-dried.

CeNP (100 mg/mL) was dispersed in 2% sodium alginate solution. The mixture was extruded into calcium chloride solution via microfluidics (10-30 min reaction), followed by 3-5 deionized water washes to remove residual reactants.

### 4.5 Fabrication of adCe-MS

CeNP-NaA core microspheres were immersed in adGel precursor and subjected to ultrasonic vibration, yielding a uniform, viscous hydrogel shell that fully enveloped each sphere. A brief, 5–10 s exposure to 284 nm UV (10 W) immediately before use drives complete in-situ cross-linking, transforming the shell into a robust, nanozyme-functionalized adhesive layer.

### 4.6 Nanoparticle characterization

The hydrodynamic diameter (D_h_) and ζ-potential of CeNP were measured using a Malvern laser granulometer (Zetasizer 3000HSA, Malvern, Britain). Each sample was measured three independent times; within every run, ten replicate readings were averaged. Morphology was visualized with a JEOL 2200FS ultra-high-resolution TEM. For SEM imaging (JEOL 7500F), CeNP, adGel and adCe-MS were lyophilized, cryo-fractured, and sputter-coated prior to observation.

### 4.7 Enzyme activity assay

RAW 264.7 cells (Procell Life, China) were exposed to graded concentrations of CeNP for 24 h. Supernatants were harvested and assayed for antioxidant capacity including superoxide dismutase (SOD) activity, hydroxyl-radical scavenging rate, and catalase (CAT) activity, using commercially available assay kits (Nanjing Jiancheng Bioengineering Institute, China) in accordance with the manufacturer's instructions. After reagent addition and color development, absorbance was read spectrophotometrically. SOD activity, hydroxyl-radical scavenging efficiency, and CAT activity were calculated from standard curves to quantify the CeNP's antioxidant potency.

### 4.8 ROS assay

RAW 264.7 cells were pre-treated for 12 h with graded concentrations of CeNP or culture medium after immersion of the hydrogel for 48 h. Oxidative stress was elicited with 0.2 μL/mL H_2_O_2_ for 1–3 h. Cells were loaded with 10 μM DCFH-DA (Solarbio, China) for 20 min. Following PBS washing, the samples were either visualized using a fluorescent microscope (Leica) or analyzed using a FACS Calibur flow cytometer (BD Fortessa, USA) in conjunction with FlowJo software.

### 4.9 Live/dead cell assay

RAW264.7 cells were cultured in 6-well plates at a density of 2 × 10^6^ per well. The cured adGel and adCe-MS were separately immersed in the medium for 48 h and configured into different concentrations of medium to treat the cells. After 24 h of culture, the cells were stained using Calcein/PI Cell Viability/Cytotoxicity Assay Kit (Beyotime Biotechnology, China) and the ratio of live cells to dead cells was observed under a fluorescence microscope.

### 4.10 Release of CeNP from adCe-MS

To quantify CeNP release, the UV-cured adCe-MS was immersed in PBS with or without collagenase (0.05 mg/mL). The system was incubated at 37 °C under constant orbital shaking at 100 rpm. At predetermined time intervals, 1 mL aliquots of the release medium were collected and immediately replaced with an equal volume of fresh PBS with or without collagenase to maintain sink conditions. Concurrently, hydrogel degradation was monitored. CeNP concentrations in the collected samples were quantified by UV–vis spectroscopy at a wavelength of 323.5 nm using an Evolution 300 spectrophotometer (Thermo Scientific). Cumulative release profiles were constructed and fitted to kinetic models to assess the hydrogel's controlled-release behavior.

### 4.11 Enzyme-linked immunosorbent assay (ELISA)

Cell supernatant was obtained after treatment of RAW264.7 cells for 24 h with medium containing varying concentrations of CeNP or medium that had been immersed in hydrogel for a duration of 48 h, and plasma was obtained by centrifugation of mouse blood samples in EDTA anticoagulated tubes for 10 min at 3000 rpm. Plasma and supernatant levels of anti-SSA antibodies (SSA Ab), anti-SSB antibodies (SSB Ab), and cytokines, including TNF-α and IL-6 were measured using mouse ELISA kits (Jingmei Biotechnology, China) according to the manufacturer's instructions.

### 4.12 Evaluation of adCe-MS adhesion to murine tongue tissue

Excised murine tongue specimens were rinsed with PBS to remove surface contaminants, after which adCe-MS was uniformly applied and photocured under UV irradiation. The samples were subsequently lyophilized and analyzed using SEM (JEOL 7500F) at various magnifications to assess the interfacial adhesion characteristics between the gel and tissue.

### 4.13 Mice and treatments

Female NOD/LtJ and C57BL/6 mice (8 weeks of age) were purchased from Jiangsu GemPharmatech Co. Ltd. and raised under specific pathogen-free conditions at the Experimental Animal Center of Nantong University. From the 13th week, the mice were coated with 10 μL of adGel, adCe-MS or PBS on the sublingual oral mucosa and cured for 5-10 s under UV light at 284 nm, 10 W power. At the end of the 4-week treatment period, mice were euthanized. The three major paired salivary glands (parotid, submandibular, and sublingual) along with major organs (heart, liver, spleen, lungs, and kidneys) were excised for subsequent analysis. The intact gland complex was utilized for ex vivo organ imaging and the calculation of the salivary gland index, whereas detailed quantitative histopathological and transcriptomic analyses were systematically performed on the submandibular glands. All animal experimental procedures were performed in accordance with the guidelines approved by the Animal Ethics Committee of Nantong University (S20250930-001).

### 4.14 Saliva collection in mice

Mouse saliva was collected by the absorbent paper method. After anesthesia, each animal received an intraperitoneal injection of pilocarpine hydrochloride (2 mg kg^−1^, MCE, USA) to stimulate salivation. Sterile absorbent paper strips (0.5 cm × 5 cm) were gently inserted into the buccal or sublingual cavity for exactly 1 min, then promptly removed. Salivary output was quantified by measuring the length of the wetted portion on each strip, allowing direct comparison among experimental groups.

### 4.15 Pathology assessment of submandibular glands

Quantitative histopathological assessment was systematically performed on the submandibular glands. The submandibular glands were fixed in 4 % paraformaldehyde-PBS, paraffin-embedded, and sectioned at 4 μm. Sections were stained with hematoxylin and eosin (H&E) and Masson for histological evaluation.

### 4.16 Flow cytometry analysis for lymphocytes in spleen

Single-cell splenocyte suspensions were prepared and washed twice in ice-cold FACS buffer (PBS + 2 % FBS) by centrifugation (800×*g*, 4 °C, 5 min). Macrophages: cells were stained for 30 min at room temperature (dark) with Fixable Viability Dye-455UV, F4/80-PerCP-Cy5.5, CD86-FITC. Treg cells were stained for 30 min at room temperature (dark) with Fixable Viability Dye-455UV, CD25-PE, FOXP3-BV421. All antibodies were from BD Biosciences or BioLegend. Data were acquired on a BD Fortessa flow cytometer and analyzed with FlowJo software.

### 4.17 Immunohistochemistry

The submandibular glands of mice were fixed in 4% paraformaldehyde, paraffin-embedded, and sectioned, followed by heat-induced antigen retrieval. Non-specific binding sites were blocked with 5% BSA, and anti-MUC1 polyclonal antibody (proteintech, China) or anti-AQP5 polyclonal antibody (proteintech, China) was incubated at 4 °C overnight. Horseradish peroxidase (HRP) -conjugated secondary antibody was then added and incubated at room temperature for 1 h. After DAB color development, MUC1 and AQP5 expression was observed under a microscope, nuclei were counterstained with hematoxylin, and sections were dehydrated, mounted, and imaged (Olympus, Japan).

### 4.18 RNA sequencing and transcriptomic analysis

Total RNA was extracted from submandibular gland tissues using Trizol reagent. RNA quality and integrity were assessed using an Agilent 2100 Bioanalyzer (Agilent Technologies, USA) and confirmed by RNase-free agarose gel electrophoresis. Gene Denovo Biotechnology Co., Ltd. (Guangzhou, China) is responsible for the complete library construction, sequencing and primary bioinformatics analysis. Differentially expressed genes (DEGs) were identified, followed by Gene Ontology (GO) and Kyoto Encyclopedia of Genes and Genomes (KEGG) pathway enrichment analyses using the company's online platform (www.genedenovo.com). Data visualization, including the generation of volcano plots, heatmaps, and radar plots for key DEGs, was conducted using tools within this platform.

### 4.19 Statistical analysis

Statistical analyses were conducted using GraphPad Prism software. All data are presented as the mean ± SD. For comparisons between two groups, two-tailed unpaired *t*-tests were performed. One-way ANOVA was utilized for multiple comparisons among more than two groups. For data that failed the normality test or exhibited unequal variance, the nonparametric Mann-Whitney *U* test was used. For all experiments, *P*-value < 0.05 was considered statistically significant.

## CRediT authorship contribution statement

**Ye Fang:** Investigation, Methodology, Writing – original draft, Writing – review & editing. **Xinyu Tao:** Writing – review & editing. **Nengjie Yang:** Writing – review & editing. **Jing Li:** Funding acquisition, Writing – review & editing. **Jun Xiao:** Funding acquisition, Writing – review & editing. **Liwei Qiu:** Funding acquisition. **Yujuan Zhu:** Conceptualization, Funding acquisition, Writing – review & editing. **Zhifeng Gu:** Conceptualization, Funding acquisition, Writing – review & editing.

## Declaration of competing interest

The authors declare that they have no known competing financial interests or personal relationships that could have appeared to influence the work reported in this paper.

## Data Availability

Data will be made available on request.
