# Supplementary material for: Microfluidic-based oral mucoadhesive nanozyme microspheres for immune modulation of xerostomia
Source: Mater Today Bio. 2026 Feb 10;37:102914. doi: 10.1016/j.mtbio.2026.102914 (PMC12924749; doi:10.1016/j.mtbio.2026.102914)
Supplement: Multimedia component 1 [file mmc1.docx]

**Supporting Information**

**Microfluidic-based Oral Mucoadhesive Nanozyme Microspheres for Immune Modulation of Xerostomia**

Ye Fang, Xinyu Tao, Nengjie Yang, Jing Li, Jun Xiao, Liwei Qiu, Yujuan Zhu^*^, Zhifeng Gu^*^

Department of Rheumatology, Research Center of Clinical Medicine, Affiliated Hospital of Nantong University, Medical School of Nantong University, Nantong University, Nantong 226001, China

Correspondence: yujuanzhu@ntu.edu.cn, guzf@ntu.edu.cn

**Methods**

**The encapsulation efficiency (EE%) and loading capacity (LC%) determination**

The encapsulation efficiency (EE%) and loading capacity (LC%) of adCe-MS were determined using the following procedures: 0.5 mL of sample was added to 0.1 M sodium citrate solution and incubated on a constant temperature shaker at 37 ℃ until the pellets were completely dissolved. CeNP extract was obtained through centrifugation. The absorbance at 323.5 nm was analyzed using a UV-Vis spectrophotometer (UV-5200, Shimadzu, Japan). The ASX content was calculated based on the standard curve Y = 0.006603X + 0.01119, which exhibited a coefficient of determination (R²) of 0.9902. The encapsulation efficiency and loading capacity were then calculated using the corresponding formulas, yielding values of 84.89% ± 2.93% and 9.33% ± 0.32%, respectively.

EE (%) = $\frac{encapulated CeNP content}{total CeNP content input}$ *100

LC (%) = $\frac{encapulated CeNP content}{weight of nanoparticles}$ *100

**Cell Counting Kit-8 (CCK-8)**

The murine macrophage cell line RAW 264.7 (ATCC TIB-71) was utilized as the experimental model. The cells were routinely cultured in high-glucose Dulbecco's modified Eagle’s medium (DMEM, GIBCO) with 10% fetal bovine serum (FBS, Excell Bio) under standard culture conditions, maintained at 37°C in a humidified atmosphere containing 5% CO_₂_. The impact of CeNP on the viability of RAW 264.7 was assessed using the Cell Counting Kit-8 (NCM Biotech, China). The viability of RAW 264.7 cells treated with CeNP was assessed using CCK-8. Cells were seeded in a 96-well plate, pre-cultured for 24 h, treated with various concentrations of CeNP solutions and incubated. After adding CCK-8 solution, absorbance at 450 nm was measured to evaluate cell proliferation rate. ${OD}_{test}$, ${OD}_{blank}$, and ${OD}_{control}$ represent the absorbance values of the test group (cells with various concentrations of CeNP solutions), the control group (cells with culture medium only), and the blank group (culture medium without cells), respectively.

Cell proliferation rate (%) = $\frac{{OD}_{test}-{OD}_{blank}}{{OD}_{control}-{OD}_{blank}}$ * 100

**Serum biochemical analysis**

Blood samples were collected via retro-orbital puncture. Following a 30-minute clotting period at room temperature, the samples were centrifuged at 3,000 × g for 15 min at 4 °C to obtain serum, which was subsequently stored at -80 °C until further analysis. Serum concentrations of ALT, AST, and BUN were determined using a fully automated biochemical analyzer (BS-240Vet, Mindray Bio-Medical Electronics Co., Ltd., China).

**Biocompatibility evaluation of the in-situ UV-curing process**

The human immortalized keratinocyte cell line HaCaT (Procell Life, China) was utilized as the experimental model in vitro cell viability assay. HaCaT keratinocytes were seeded in culture plates and allowed to adhere overnight. Prior to irradiation, the culture medium was replaced with PBS. The cells were then subjected to UV light (284 nm, 10 W) for 0, 5, or 10 s. After 24 h of culture, the cells were stained using Calcein/PI Cell Viability/Cytotoxicity Assay Kit (Beyotime Biotechnology, China) according to the manufacturer’s protocol, and visualized under a fluorescence microscope.

To evaluate the safety of the in-situ UV-curing procedure in vivo, 12-week-old female C57BL/6 mice were anesthetized, and their oral cavities were gently opened to fully expose the buccal mucosa. The target areas were then irradiated with UV light (284 nm, 10 W) for 0, 5, or 10 s using a fixed probe positioned at a consistent distance. Immediately after irradiation, the mice were euthanized, and the irradiated mucosal tissues were harvested. Tissue samples were fixed, paraffin-embedded, sectioned, and stained with H&E for histopathological analysis.

**Preparation of Cy5-labeled CeNP**

CeNP was dispersed in 10 mL PBS at a concentration of 6 mg/mL. Sulfo-Cy5 (2 mg/mL, MCE, USA) methanol solution was added dropwise to the CeNP suspension under continuous stirring at 300 rpm. Unbound dye was removed by repeated centrifugation (15,000 × g, 30 min) until the supernatant was clear and non-fluorescent. The pellet was resuspended in PBS to obtain the Cy5-CeNP conjugate, which was stored at 4°C in the dark.

**Ex vivo imaging**

Fluorescent formulations with equivalent total Cy5 content were prepared. Cy5-loaded sodium alginate microspheres (Cy5-MS) and Cy5-CeNP-loaded sodium alginate microspheres (Cy5-CeNP-MS) were fabricated by incorporating the dye or pre-labeled Cy5-CeNP into a 2% sodium alginate solution, followed by microfluidic electrospray into calcium chloride solution. Cy5-adCe-MS were obtained by further encapsulating Cy5-CeNP-MS in adGel. BALB/c mice (n = 3 per group) were administered Cy5-MS or Cy5-CeNP-MS in PBS via oral gavage (100 µL/mouse); or Cy5-adCe-MS via topical application to the oral mucosa (10 µL/mouse). At 6, 12, and 24 h post-administration, major organs—including the heart, liver, spleen, lungs, and kidneys, as well as the entire salivary gland tissue complex (encompassing the parotid, submandibular, and sublingual glands)—were harvested for ex vivo fluorescence imaging (IVIS Spectrum, PerkinElmer, USA). Fluorescence intensity was quantified by defining regions of interest (ROIs), with data expressed as mean radiant efficiency.

**Figures**

**
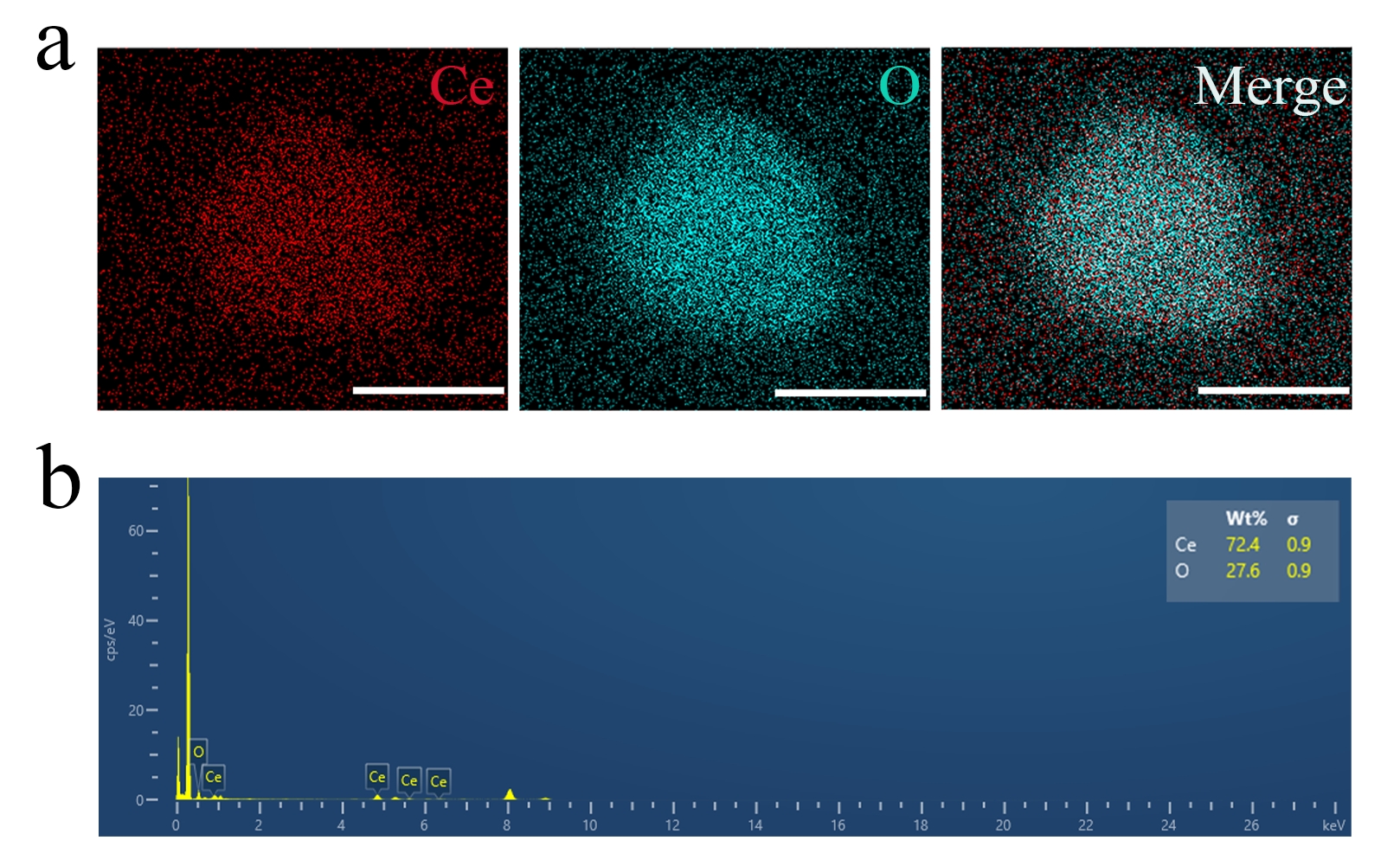
**

**Figure S1. Elemental composition analysis of CeNP. a,** Elemental mapping showing the spatial distribution of Ce (red) and O (green). Scale bar, 50 nm. **b,** EDS spectrum confirming the presence of Ce and O elements.

**
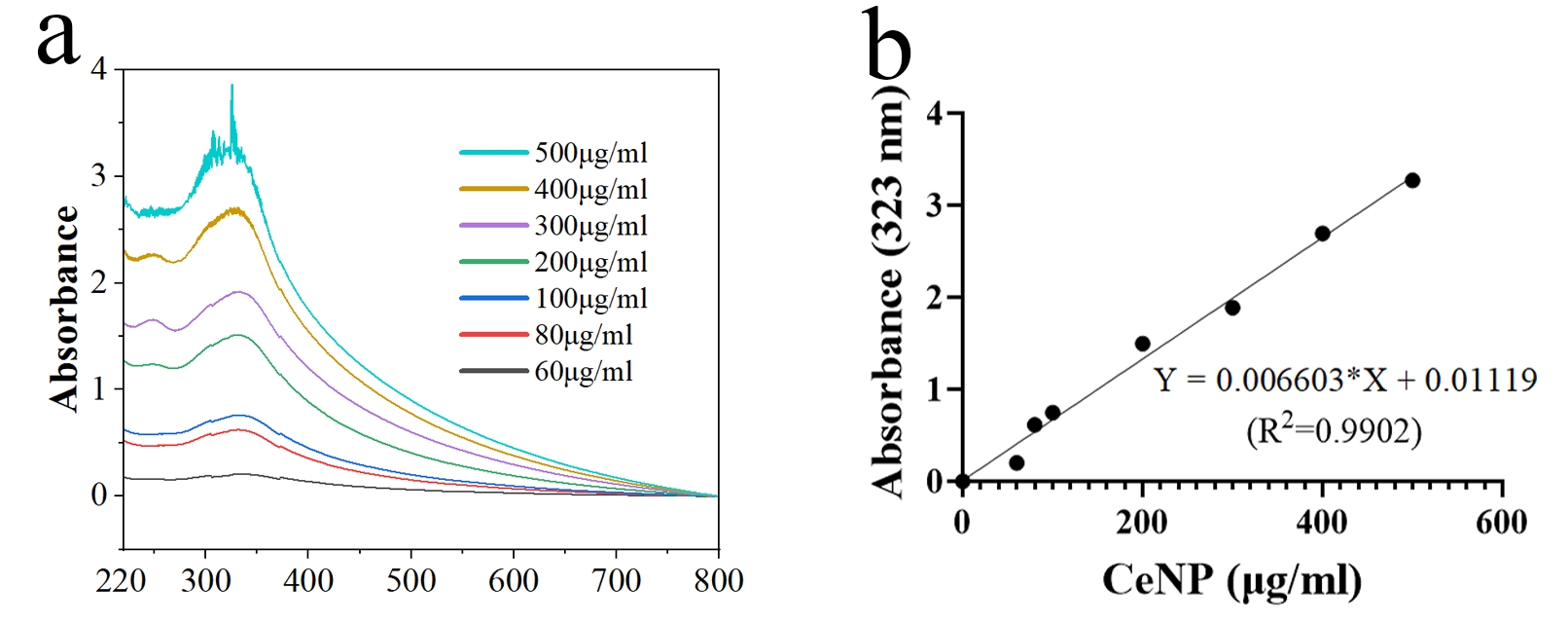
**

**Figure S2. Drug loading performance of the microspheres.** The encapsulation efficiency (EE%) and loading capacity (LC%) was calculated as described in Methods.


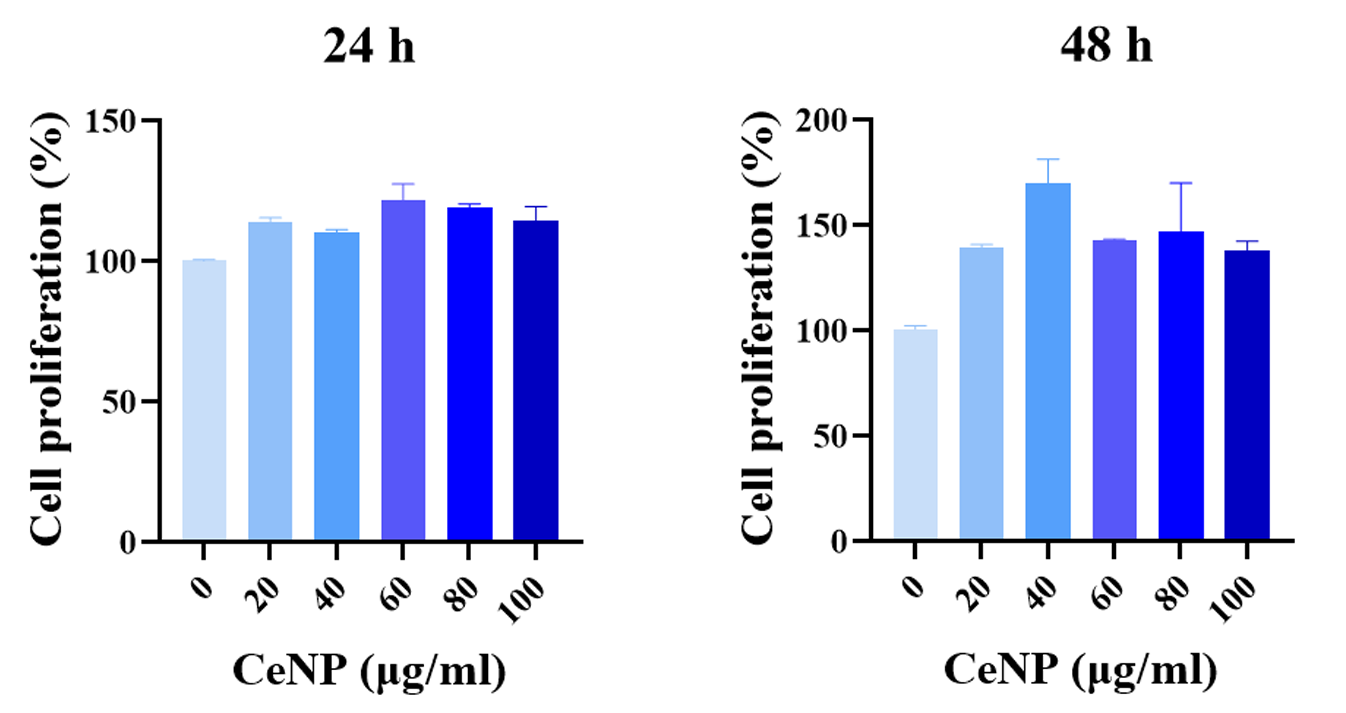


**Figure S3. Cytocompatibility assessment of CeNP.** Cell proliferation rate of RAW 264.7 cells after incubation with various concentrations of CeNP for 24 h and 48 h, as determined by CCK-8 assay. Data are presented as mean ± SD (n = 3).

**
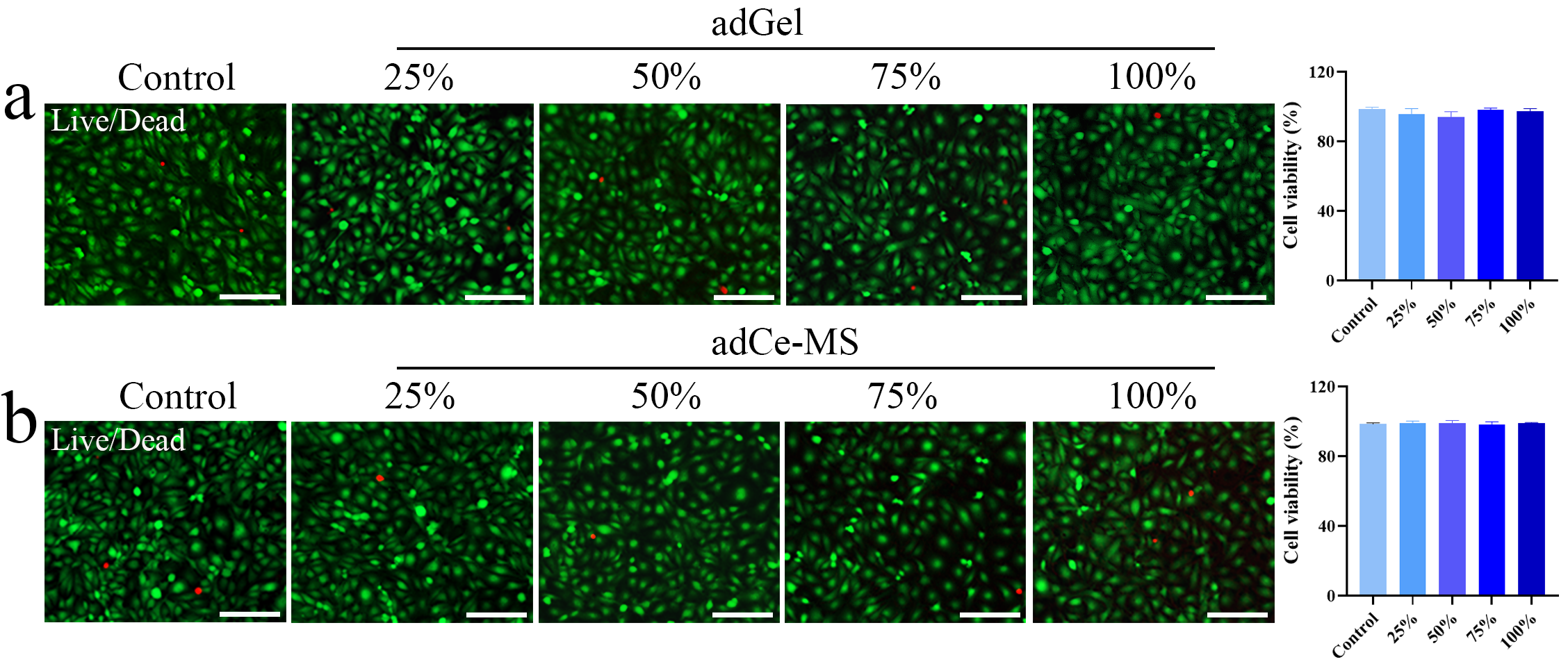
**

**Figure S4. Biocompatibility** **evaluation of adGel and adCe-MS. a-b,** Viability of macrophages exposed to adGel and adCe-MS was analyzed by staining live cells with Calcein AM (green) and dead cells with Propidium Iodide (PI, red). Scale bar, 100 μm. Data are presented as mean ± SD (n = 3).

**
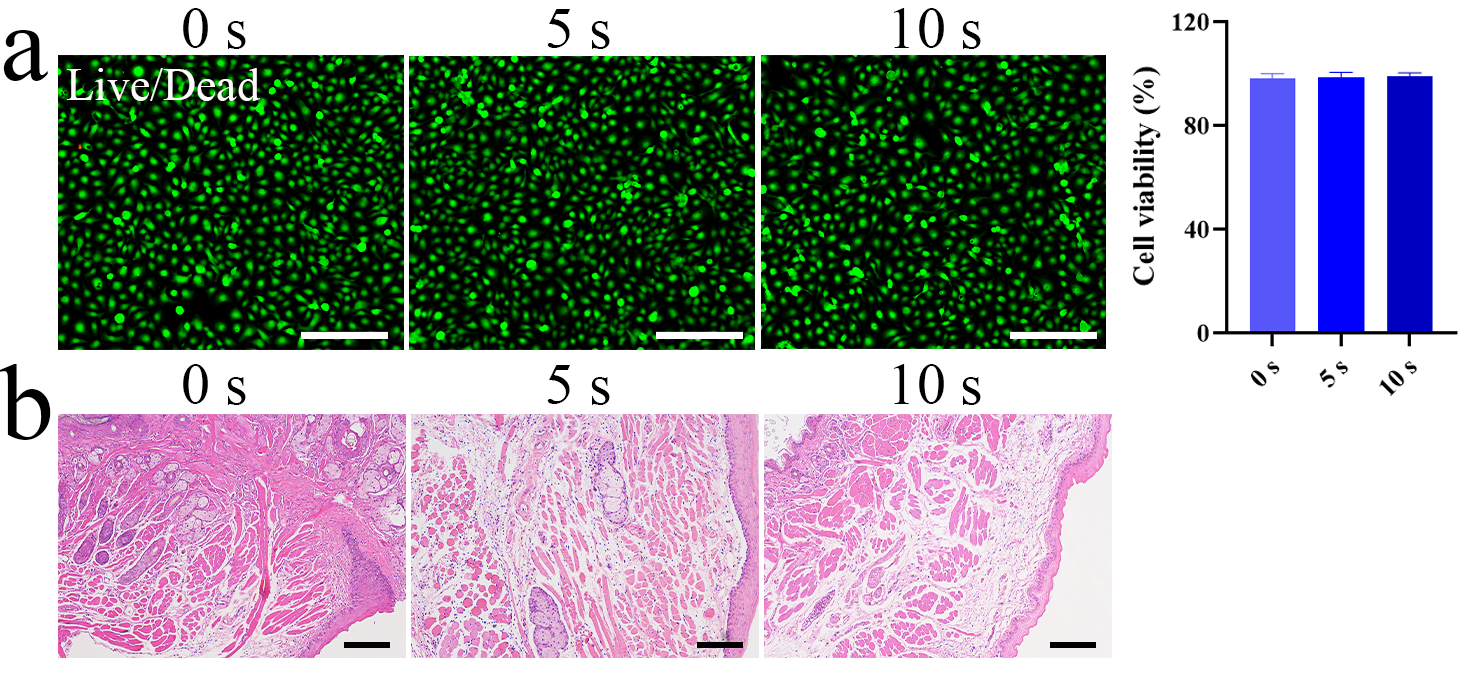
**

**Figure S5.** **Biocompatibility evaluation of the in-situ UV-curing process. a,** Viability of HaCaT keratinocytes following UV irradiation (284 nm, 10 W) for the indicated durations (0, 5, 10 s) was analyzed by staining live cells with Calcein AM (green) and dead cells with PI (red). Scale bar, 100 μm. **b,** H&E staining of murine buccal mucosa after exposure to the same UV irradiation protocol. Scale bar, 100 μm. Data are presented as mean ± SD (n = 3).


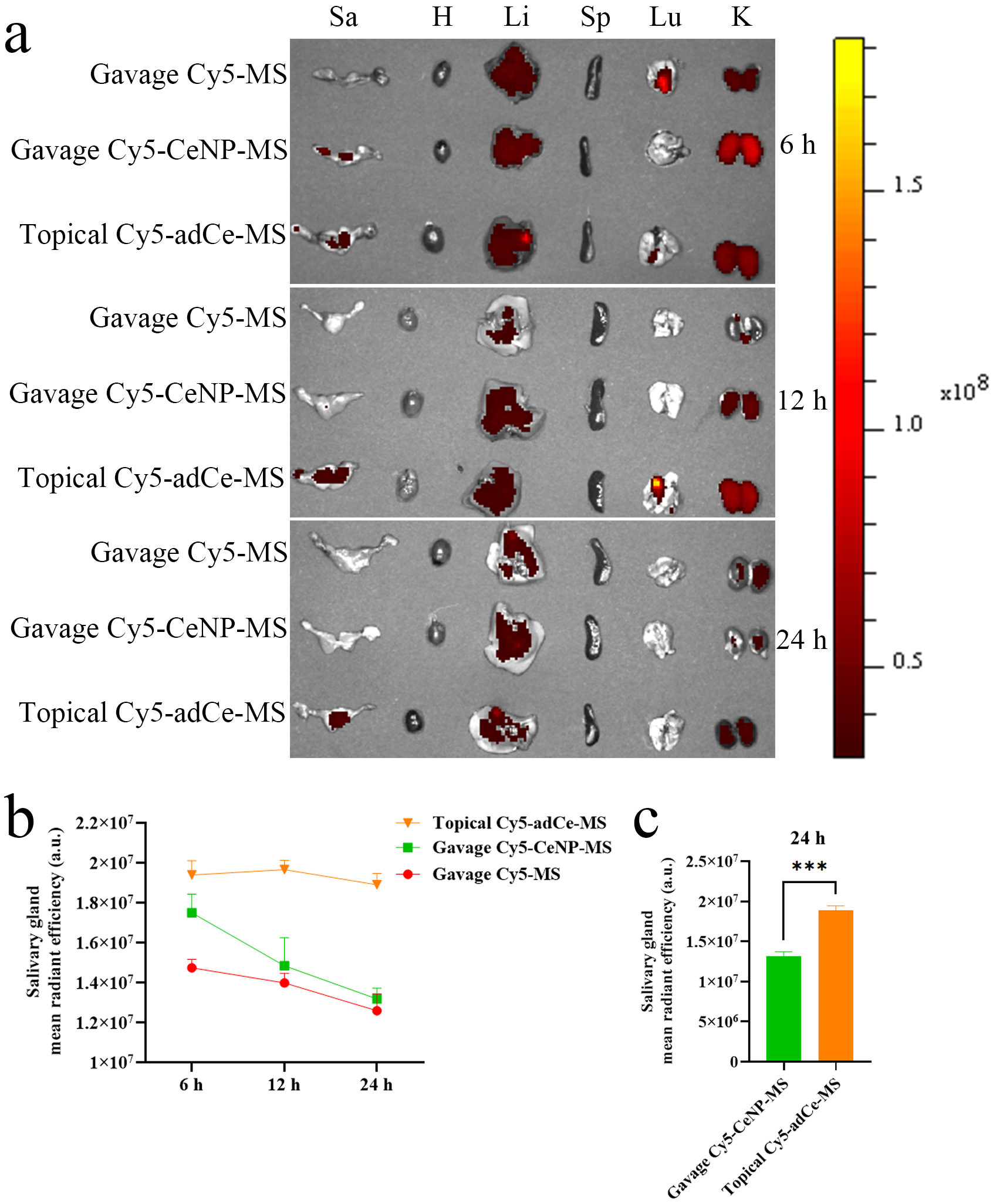


**Figure S6.** **Biodistribution and pharmacokinetics of Cy5-labeled formulations after topical versus systemic administration. a,** Ex vivo fluorescence images of major organs harvested at 6, 12, and 24 h post-administration. Formulations were delivered either by oral gavage (Cy5-loaded sodium alginate microspheres, Cy5-MS; Cy5-CeNP-loaded sodium alginate microspheres, Cy5-CeNP-MS) or topical application to the oral mucosa (Cy5-adCe-MS). Sa, salivary gland; H, heart; Li, liver; Sp, spleen; Lu, lung; K, kidney. **b,** Quantitative analysis of fluorescence intensity in salivary glands over time (n = 3). Data are expressed as the mean radiant efficiency in units of [p/s/cm²/sr] / [µW/cm²]. **c,** Comparison of salivary gland fluorescence intensity at 24 h between the Cy5-CeNP-MS (gavage) and Cy5-adCe-MS (topical) groups (n = 3). Data are represented as means ± SD. * *P* < 0.05, ** *P* < 0.01, *** *P* < 0.001.


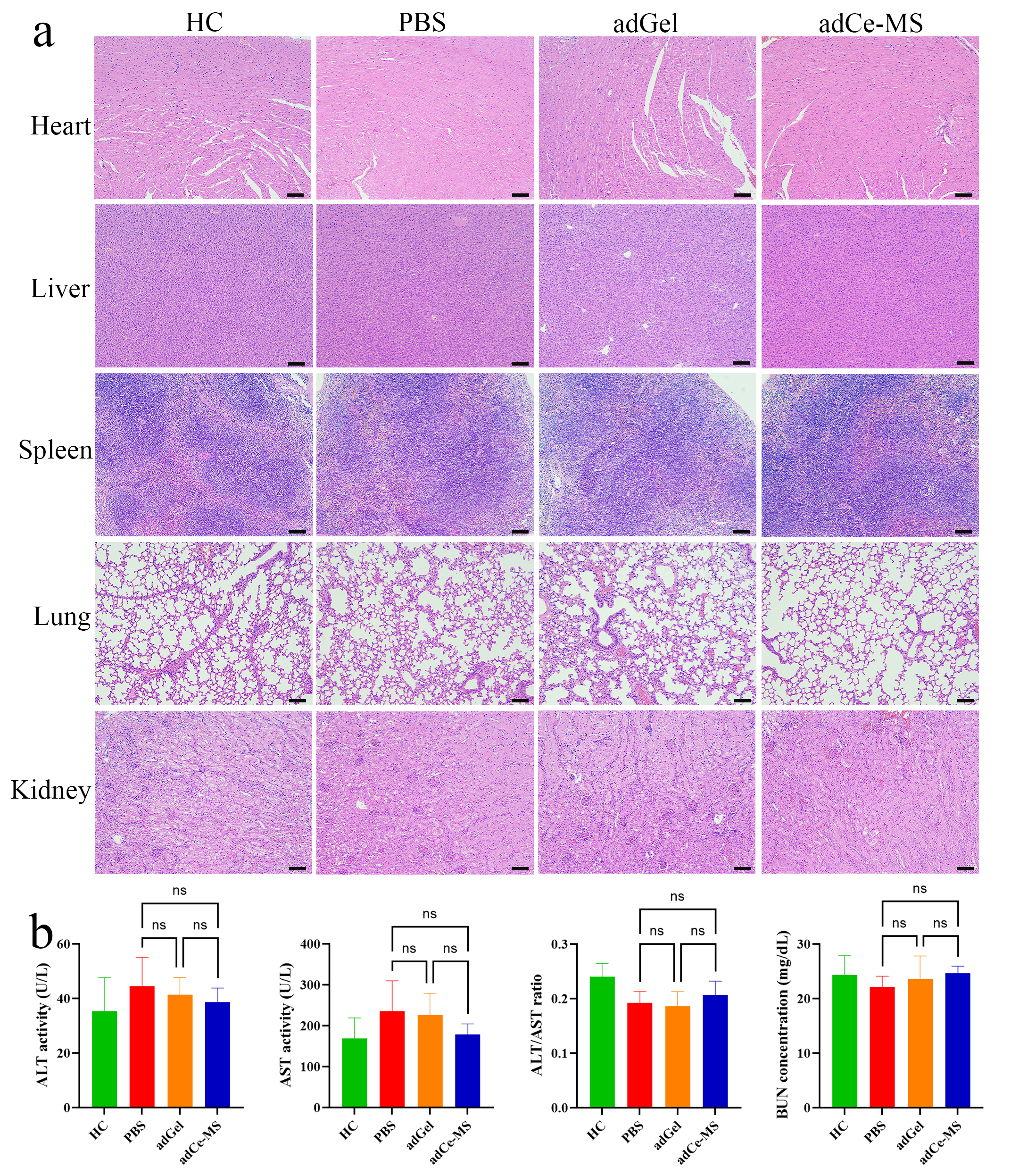


**Figure S7. Evaluation of biocompatibility after four weeks of treatment. a,** H&E staining of main organs of xerostomic mice. Scale bar, 200 μm. **b,** Serum levels of alanine aminotransferase (ALT), aspartate aminotransferase (AST), blood urea nitrogen (BUN) and the ALT/AST ratio across groups. All the values are represented as the means ± SD (n = 4). No significant differences.

**
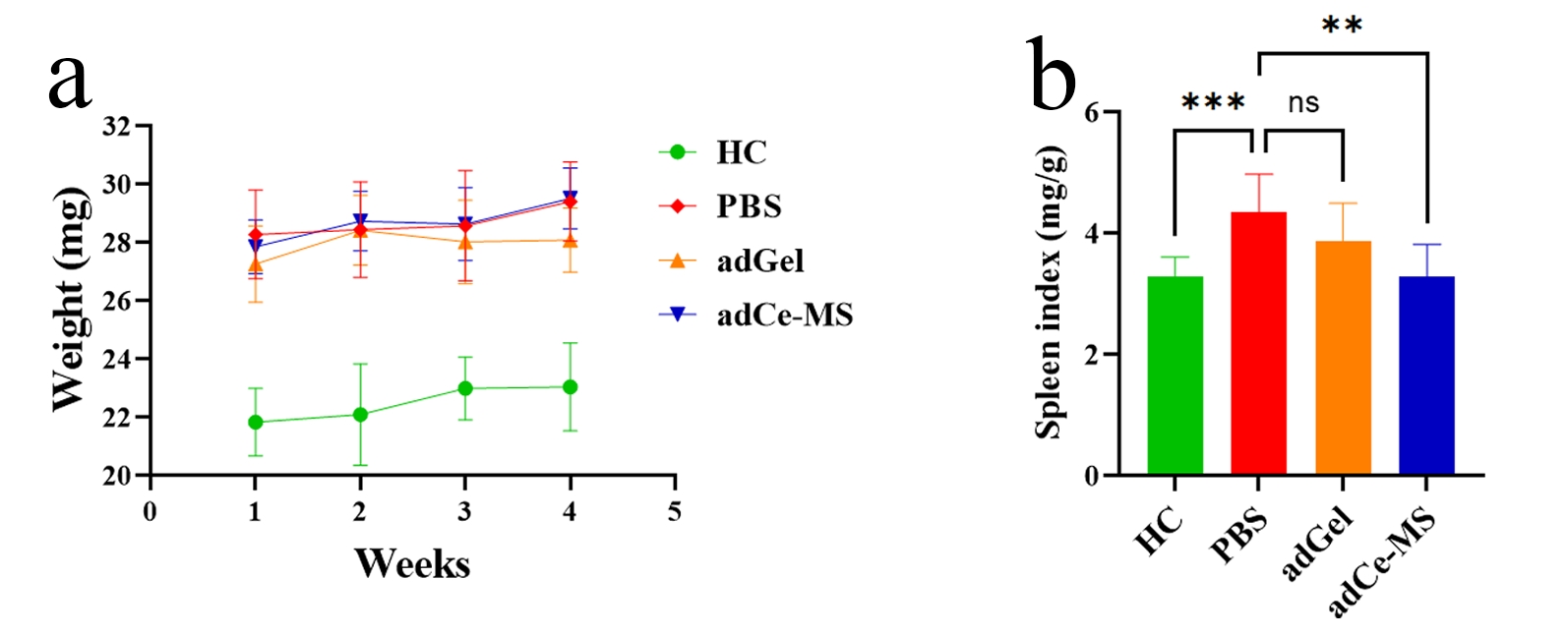
**

**Figure S8. Systemic safety assessment of different treatments in xerostomic mice. a,** Weight changes of xerostomic mice with different treatments (n = 8). **b,** Spleen index of xerostomic mice with different treatments. All the values are represented as the means ± SD (n = 8). Statistical significance was indicated as * *P*<0.05, ** *P*<0.01, *** *P*<0.001.

**
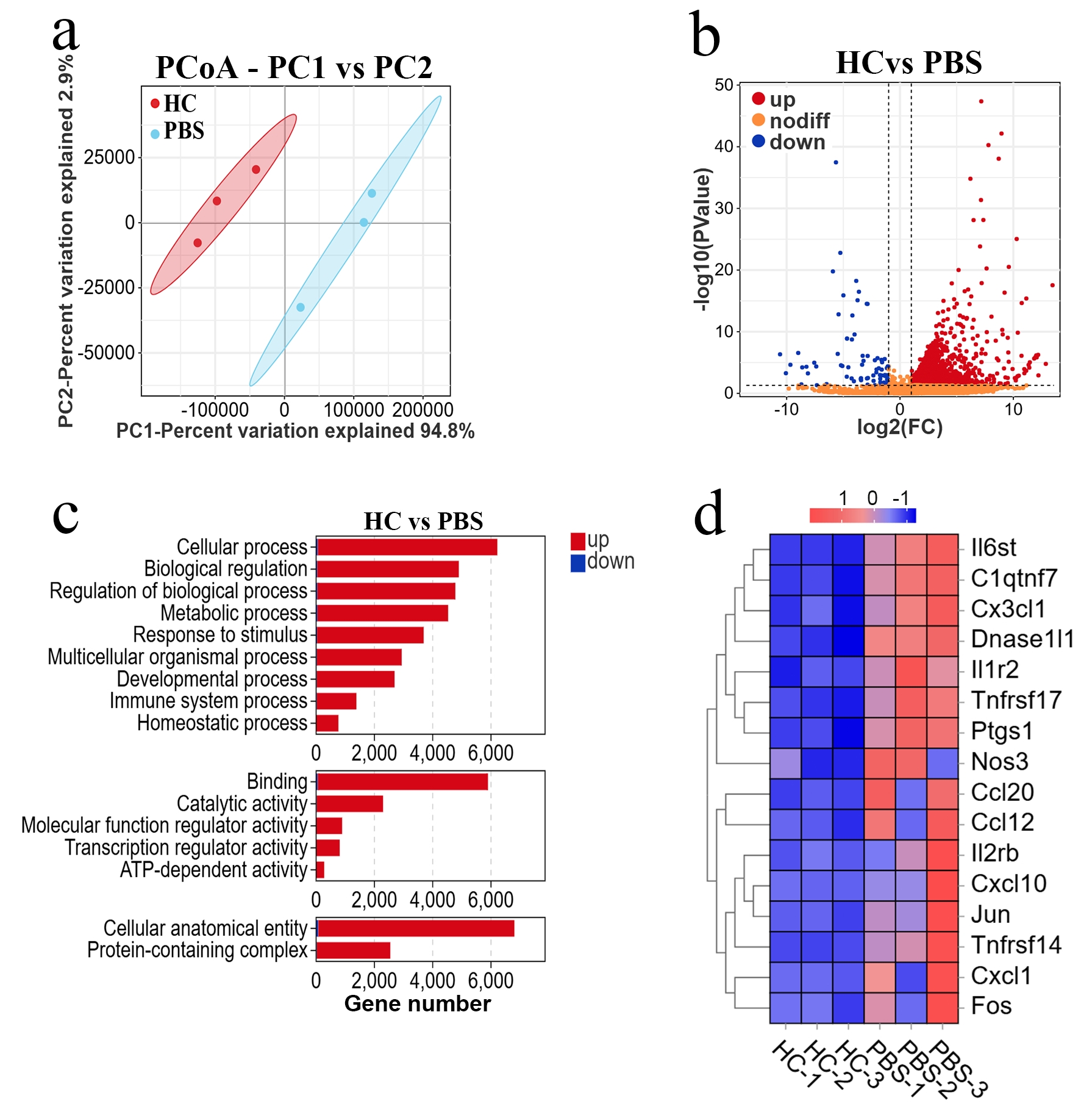
Figure S9. Transcriptomic profiling of submandibular glands in xerostomic mice.** **a,** PCoA of gene expression profiles comparing the healthy control and xerostomic groups. **b,** Volcano plots of DEGs between the healthy control and xerostomic groups. **c,** Abundant subcategories enriched by DEGs in the biological process, molecular function, and cellular component. **d,** Heatmap displaying the expression patterns of inflammation-related genes.

**
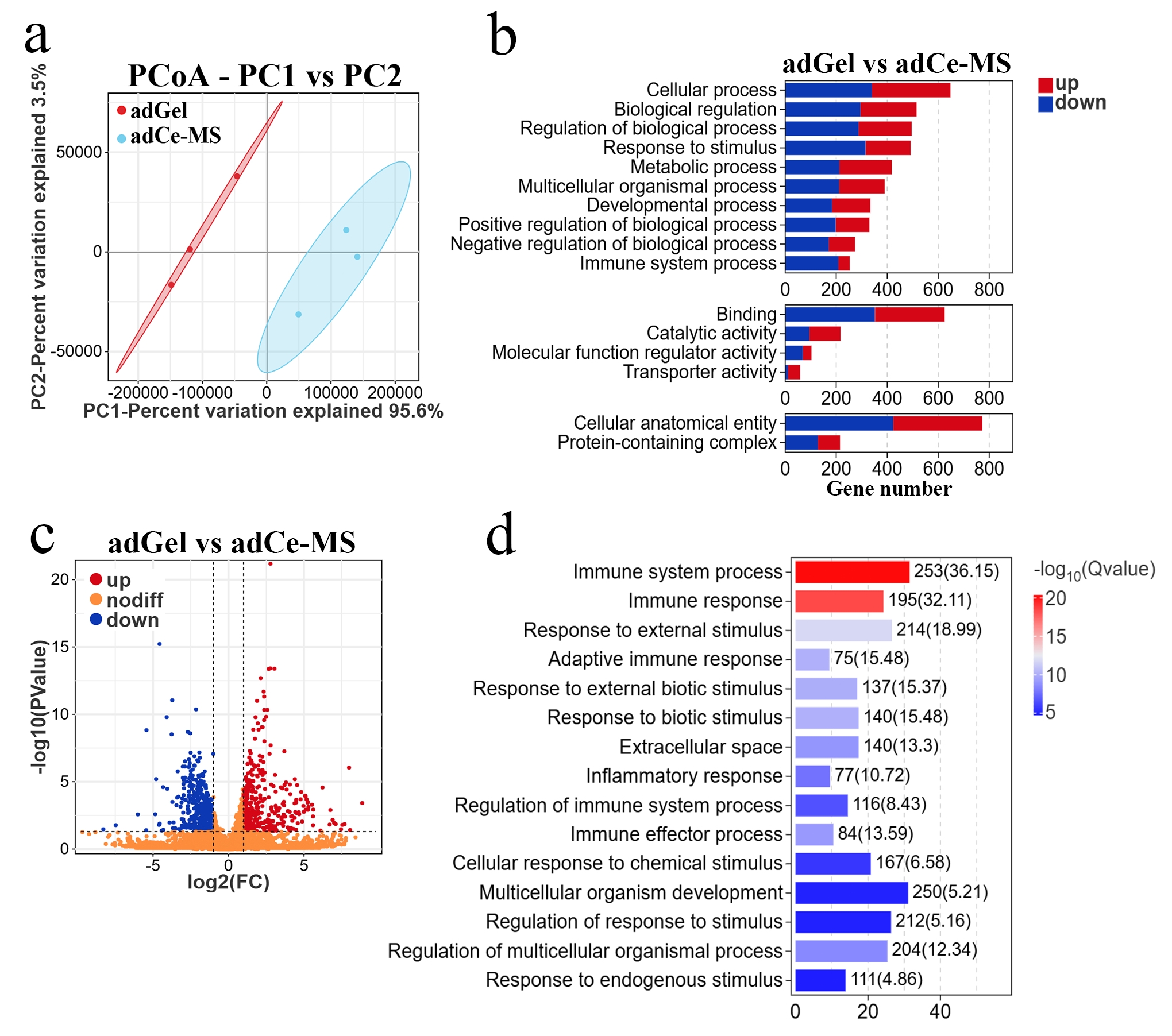
**

**Figure S10. Comparative transcriptomic analysis of adGel versus adCe-MS treatment in xerostomic mice.** **a,** PCoA of gene expression profiles between the adGel- and adCe-MS-treated groups. **b,** Abundant subcategories enriched by DEGs in the biological process
